# Supplementary material for: Moderation of Structural DNA Properties by Coupled Dinucleotide Contents in Eukaryotes
Source: Genes (Basel). 2023 Mar 20;14(3):755. doi: 10.3390/genes14030755 (PMC10048725; doi:10.3390/genes14030755)
Supplement: Supplementary file 1 [file genes-14-00755-s001.zip › genes-2270988-supplementary.pdf]

Table S1: Accession numbers of added genomes

Species	Accession Numbers
Gorilla gorilla	NC_044602.1 - NC_044625.1
Pongo abelii	NC_036903.1 - NC_036926.1
Microcebus Murinus	NC_033660.1 - NC_033692.1
Microtus ochrogaster	NC_022009.1 - NC_022036.1
Ochotona princeps	NC_050536.1 - NC_050568.1
Macaca mulatta	NC_027893.1 - NC_027914.1

Table S2: Correlation values and significance levels between dinucleotide pairs: Pearson correlation values of normalized chromosomal dinucleotide contents for empirical content and model contents (model1: binomial, model2: Chargaff binomial). The significance levels are calculated by dividing the difference between empirical and model values by the respective error of the correlation value.

dinucleotides		empirical correlation value	model1 correlation value	model1 significance level	model2 correlation value	model2 significance level
TT	AA	1.00	-0.01 ± 0.10	10.57	0.37 ± 0.10	6.26
AT	AA	-0.56	0.01 ± 0.10	-5.50	0.01 ± 0.10	-5.66
AT	TT	-0.56	0.03 ± 0.10	-5.81	0.02 ± 0.10	-5.86
TA	AA	-0.63	-0.02 ± 0.12	-5.32	-0.00 ± 0.11	-5.53
TA	TT	-0.64	-0.01 ± 0.11	-5.48	0.01 ± 0.10	-6.47
TA	AT	0.69	0.01 ± 0.07	9.10	0.05 ± 0.13	4.98
AC	AA	-0.36	0.03 ± 0.09	-4.47	0.04 ± 0.14	-2.89
AC	TT	-0.36	-0.00 ± 0.13	-2.76	-0.04 ± 0.13	-2.51
AC	AT	0.21	-0.02 ± 0.10	2.39	-0.07 ± 0.08	3.49
AC	TA	0.34	0.03 ± 0.11	2.83	-0.02 ± 0.11	3.25
CA	AA	-0.18	0.04 ± 0.10	-2.15	0.05 ± 0.11	-2.00
CA	TT	-0.18	0.08 ± 0.09	-2.71	0.05 ± 0.12	-1.88
CA	AT	-0.44	0.06 ± 0.11	-4.62	-0.05 ± 0.12	-3.24
CA	TA	-0.31	-0.04 ± 0.1	-2.69	-0.02 ± 0.09	-3.07
CA	AC	-0.05	0.00 ± 0.12	-0.41	0.18 ± 0.11	-2.13
TG	AA	-0.18	0.01 ± 0.10	-1.98	0.08 ± 0.13	-1.97
TG	TT	-0.18	0.03 ± 0.07	-2.97	0.06 ± 0.10	-2.46
TG	AT	-0.44	0.00 ± 0.05	-9.19	-0.06 ± 0.12	-3.19
TG	TA	-0.3	0.04 ± 0.10	-3.59	-0.06 ± 0.14	-1.69
TG	AC	-0.04	-0.01 ± 0.08	-0.49	0.22 ± 0.14	-1.88
TG	CA	1.0	-0.02 ± 0.06	17.64	0.43 ± 0.10	5.63
GT	AA	-0.36	-0.00 ± 0.06	-5.52	0.04 ± 0.11	-3.49
GT	TT	-0.36	0.05 ± 0.05	-8.03	0.05 ± 0.10	-4.03
GT	AT	0.21	0.01 ± 0.08	2.59	-0.01 ± 0.14	1.59
GT	TA	0.34	-0.03 ± 0.08	4.56	-0.03 ± 0.13	2.78
GT	AC	0.99	-0.01 ± 0.07	15.01	0.43 ± 0.11	5.18
GT	CA	-0.05	0.03 ± 0.08	-1.06	0.16 ± 0.10	-2.24
GT	TG	-0.04	-0.01 ± 0.07	-0.53	0.18 ± 0.09	-2.60
AG	AA	-0.15	0.03 ± 0.08	-2.30	-0.01 ± 0.12	-1.15
AG	TT	-0.15	-0.03 ± 0.08	-1.41	-0.06 ± 0.15	-0.63
AG	AT	-0.53	-0.00 ± 0.11	-4.83	0.04 ± 0.12	-4.77

AG	TA	-0.28	-0.02 ± 0.06	-4.47	0.02 ± 0.14	-2.14
AG	AC	-0.56	0.02 ± 0.09	-6.21	-0.15 ± 0.17	-2.45
AG	CA	0.59	-0.02 ± 0.09	7.13	-0.22 ± 0.15	5.37
AG	TG	0.59	0.03 ± 0.05	11.81	-0.26 ± 0.12	7.05
AG	GT	-0.56	0.01 ± 0.08	-7.28	-0.15 ± 0.09	-4.81
GA	AA	-0.11	-0.02 ± 0.07	-1.10	-0.04 ± 0.12	-0.51
GA	TT	-0.1	-0.05 ± 0.10	-0.54	-0.02 ± 0.07	-1.10
GA	AT	0.27	-0.03 ± 0.07	4.54	0.05 ± 0.10	2.24
GA	TA	-0.19	0.01 ± 0.05	-3.72	0.00 ± 0.11	-1.73
GA	AC	0.04	0.02 ± 0.05	0.37	-0.17 ± 0.11	1.98
GA	CA	-0.57	-0.01 ± 0.12	-4.63	-0.17 ± 0.08	-4.89
GA	TG	-0.57	0.01 ± 0.06	-9.61	-0.21 ± 0.05	-6.78
GA	GT	0.03	-0.02 ± 0.07	0.80	-0.12 ± 0.10	1.54
GA	AG	-0.17	-0.04 ± 0.04	-3.08	0.22 ± 0.10	-4.04
TC	AA	-0.1	-0.01 ± 0.05	-1.80	-0.08 ± 0.13	-0.19
TC	TT	-0.1	0.02 ± 0.07	-1.91	-0.05 ± 0.15	-0.33
TC	AT	0.27	-0.02 ± 0.08	3.56	-0.02 ± 0.11	2.78
TC	TA	-0.19	0.01 ± 0.1	-2.06	0.05 ± 0.12	-1.94
TC	AC	0.03	0.04 ± 0.11	-0.05	-0.12 ± 0.12	1.28
TC	CA	-0.57	0.01 ± 0.10	-5.89	-0.19 ± 0.11	-3.34
TC	TG	-0.57	0.00 ± 0.07	-8.35	-0.19 ± 0.14	-2.77
TC	GT	0.03	-0.05 ± 0.07	1.19	-0.11 ± 0.13	1.09
TC	AG	-0.17	0.03 ± 0.07	-2.90	0.18 ± 0.18	-1.95
TC	GA	1.0	0.01 ± 0.07	14.56	0.46 ± 0.11	5.03
CT	AA	-0.15	0.01 ± 0.06	-2.93	-0.05 ± 0.11	-0.96
CT	TT	-0.15	0.02 ± 0.10	-1.66	-0.04 ± 0.10	-1.04
CT	AT	-0.53	0.03 ± 0.12	-4.72	0.07 ± 0.10	-6.12
CT	TA	-0.28	0.08 ± 0.07	-5.29	-0.00 ± 0.13	-2.10
CT	AC	-0.55	-0.03 ± 0.12	-4.50	-0.16 ± 0.13	-3.05
CT	CA	0.59	0.05 ± 0.09	6.09	-0.18 ± 0.15	5.19
CT	TG	0.59	0.01 ± 0.07	7.74	-0.23 ± 0.08	9.84
CT	GT	-0.56	0.00 ± 0.09	-6.48	-0.20 ± 0.05	-7.85
CT	AG	1.0	-0.00 ± 0.10	9.83	0.49 ± 0.08	6.44
CT	GA	-0.17	0.03 ± 0.08	-2.38	0.23 ± 0.05	-7.66
CT	TC	-0.17	-0.03 ± 0.11	-1.35	0.23 ± 0.15	-2.78
CC	AA	0.28	-0.02 ± 0.06	4.78	0.11 ± 0.19	0.86
CC	TT	0.28	-0.04 ± 0.14	2.29	0.12 ± 0.17	0.91
CC	AT	-0.26	-0.00 ± 0.08	-3.45	-0.09 ± 0.17	-1.03
CC	TA	-0.1	-0.01 ± 0.13	-0.72	-0.08 ± 0.19	-0.12
CC	AC	-0.63	-0.02 ± 0.12	-5.11	0.30 ± 0.17	-5.44
CC	CA	0.34	0.02 ± 0.06	5.52	0.30 ± 0.17	0.23
CC	TG	0.34	-0.03 ± 0.04	8.42	0.37 ± 0.08	-0.41
CC	GT	-0.62	0.02 ± 0.11	-5.94	0.34 ± 0.11	-9.05
CC	AG	0.43	-0.04 ± 0.10	4.47	-0.37 ± 0.12	6.75
CC	GA	-0.57	0.03 ± 0.13	-4.66	-0.31 ± 0.08	-3.23
CC	TC	-0.57	-0.02 ± 0.08	-6.84	-0.32 ± 0.22	-1.12
CC	CT	0.42	0.03 ± 0.12	3.22	-0.37 ± 0.09	9.05

GG	AA	0.27	0.01 ± 0.06	4.44	0.12 ± 0.18	0.86
GG	TT	0.28	-0.00 ± 0.06	4.81	0.13 ± 0.17	0.89
GG	AT	-0.26	0.03 ± 0.05	-6.17	-0.10 ± 0.15	-1.08
GG	TA	-0.1	0.02 ± 0.07	-1.60	-0.05 ± 0.18	-0.28
GG	AC	-0.63	-0.03 ± 0.08	-7.39	0.27 ± 0.14	-6.40
GG	CA	0.34	-0.03 ± 0.07	5.21	0.30 ± 0.17	0.26
GG	TG	0.34	0.02 ± 0.06	5.29	0.40 ± 0.08	-0.79
GG	GT	-0.63	0.01 ± 0.08	-7.58	0.31 ± 0.10	-9.02
GG	AG	0.42	-0.03 ± 0.09	5.04	-0.38 ± 0.09	9.18
GG	GA	-0.57	-0.02 ± 0.07	-8.03	-0.30 ± 0.10	-2.75
GG	TC	-0.57	0.01 ± 0.08	-7.66	-0.31 ± 0.21	-1.23
GG	CT	0.42	0.05 ± 0.07	5.25	-0.40 ± 0.10	8.06
GG	CC	1.0	0.02 ± 0.10	9.82	0.76 ± 0.05	4.59
GC	AA	-0.09	0.03 ± 0.14	-0.84	-0.09 ± 0.19	-0.01
GC	TT	-0.09	-0.01 ± 0.06	-1.34	-0.09 ± 0.15	0.03
GC	AT	0.03	-0.06 ± 0.09	1.05	0.10 ± 0.14	-0.48
GC	TA	0.08	-0.05 ± 0.09	1.58	0.06 ± 0.16	0.11
GC	AC	0.26	0.02 ± 0.12	1.95	-0.29 ± 0.16	3.37
GC	CA	-0.02	-0.03 ± 0.10	0.13	-0.28 ± 0.16	1.67
GC	TG	-0.01	0.03 ± 0.06	-0.72	-0.35 ± 0.09	3.62
GC	GT	0.26	-0.01 ± 0.06	4.59	-0.32 ± 0.10	5.64
GC	AG	-0.13	0.03 ± 0.06	-2.56	0.33 ± 0.12	-3.98
GC	GA	0.01	-0.02 ± 0.07	0.44	0.30 ± 0.12	-2.41
GC	TC	0.01	0.01 ± 0.11	0.07	0.29 ± 0.21	-1.29
GC	CT	-0.13	-0.02 ± 0.08	-1.35	0.34 ± 0.06	-7.29
GC	CC	-0.7	0.01 ± 0.06	-11.74	-0.61 ± 0.05	-1.96
GC	GG	-0.7	0.00 ± 0.05	-13.49	-0.59 ± 0.05	-2.14
CG	AA	-0.03	0.01 ± 0.07	-0.59	-0.11 ± 0.17	0.45
CG	TT	-0.03	-0.00 ± 0.10	-0.33	-0.08 ± 0.13	0.38
CG	AT	0.49	-0.03 ± 0.12	4.53	0.09 ± 0.15	2.66
CG	TA	0.26	0.04 ± 0.1	2.21	0.08 ± 0.16	1.09
CG	AC	0.56	0.06 ± 0.09	5.49	-0.30 ± 0.15	5.8
CG	CA	-0.75	0.02 ± 0.08	-9.34	-0.30 ± 0.16	-2.76
CG	TG	-0.75	0.01 ± 0.11	-7.00	-0.38 ± 0.08	-4.33
CG	GT	0.56	-0.02 ± 0.06	9.68	-0.31 ± 0.11	7.58
CG	AG	-0.8	0.03 ± 0.08	-10.05	0.34 ± 0.13	-8.80
CG	GA	0.57	0.00 ± 0.07	8.41	0.29 ± 0.07	3.84
CG	TC	0.57	0.00 ± 0.06	8.85	0.32 ± 0.15	1.71
CG	CT	-0.8	0.02 ± 0.09	-8.88	0.36 ± 0.09	-12.75
CG	CC	-0.82	-0.03 ± 0.10	-7.65	-0.59 ± 0.07	-3.23
CG	GG	-0.82	-0.03 ± 0.10	-8.05	-0.59 ± 0.05	-4.96

Table S3: Correlation values between dinucleotide content and attributes: Pearson correlation values between normalized chromosomal dinucleotide contents and attributes (genes: content genes, CDS: content of CDS, G+C: G+C content, enhancer: normalized enhancer counts, see 2.2 for details) for empirical dinucleotide contents and model contents (Chargaff binomial model).

dinucleotide	correlated	empirical	Chargaf model

	attribute	correlation value	Correlation value	
AA	genes	-0.009	0.014	± 0.027
AA	CDS	-0.135	0.014	± 0.037
AA	enhancer	0.004	-0.004	± 0.084
AA	G+C	-0.300	0.006	± 0.025
AA	length	0.030	-0.004	± 0.012
TT	genes	-0.007	-0.003	± 0.023
TT	CDS	-0.134	-0.001	± 0.028
TT	enhancer	0.009	0.007	± 0.080
TT	G+C	-0.300	0.010	± 0.017
TT	length	0.031	-0.002	± 0.012
AT	genes	0.007	-0.012	± 0.029
AT	CDS	0.367	-0.015	± 0.039
AT	enhancer	-0.093	-0.038	± 0.058
AT	G+C	-0.009	-0.019	± 0.021
AT	length	-0.200	0.003	± 0.017
TA	genes	-0.073	0.002	± 0.014
TA	CDS	0.007	0.000	± 0.022
TA	enhancer	-0.019	-0.003	± 0.055
TA	G+C	0.021	-0.007	± 0.024
TA	length	-0.016	0.004	± 0.011
AC	genes	0.214	0.000	± 0.033
AC	CDS	0.289	-0.001	± 0.039
AC	enhancer	-0.055	-0.011	± 0.075
AC	G+C	0.178	0.005	± 0.028
AC	length	-0.194	-0.002	± 0.013
CA	genes	-0.072	0.008	± 0.018
CA	CDS	-0.396	0.013	± 0.026
CA	enhancer	0.081	-0.031	± 0.084
CA	G+C	-0.017	0.012	± 0.027
CA	length	0.198	-0.004	± 0.013
TG	genes	-0.072	0.003	± 0.020
TG	CDS	-0.395	0.007	± 0.021
TG	enhancer	0.082	-0.006	± 0.074
TG	G+C	-0.016	-0.000	± 0.031
TG	length	0.197	-0.004	± 0.011
GT	genes	0.211	0.013	± 0.019
GT	CDS	0.283	0.020	± 0.032
GT	enhancer	-0.060	-0.022	± 0.070
GT	G+C	0.170	0.009	± 0.035
GT	length	-0.193	-0.009	± 0.013
AG	genes	-0.148	-0.005	± 0.021
AG	CDS	-0.406	-0.007	± 0.027
AG	enhancer	0.064	0.014	± 0.054
AG	G+C	0.155	-0.004	± 0.024
AG	length	0.286	0.003	± 0.012
GA	genes	0.181	0.007	± 0.023
GA	CDS	0.593	0.014	± 0.032

GA	enhancer	-0.087	-0.008	± 0.098
GA	G+C	0.322	0.004	± 0.023
GA	length	-0.240	-0.006	± 0.010
TC	genes	0.179	-0.008	± 0.022
TC	CDS	0.592	-0.009	± 0.030
TC	enhancer	-0.097	-0.019	± 0.084
TC	G+C	0.321	-0.003	± 0.030
TC	length	-0.240	0.001	0.011
CT	genes	-0.147	0.009	0.020
CT	CDS	-0.403	-0.007	0.030
CT	enhancer	0.063	-0.012	0.082
CT	G+C	0.160	-0.005	0.023
CT	length	0.284	0.003	0.015
CC	genes	-0.300	0.000	0.030
CC	CDS	-0.613	-0.008	0.023
CC	enhancer	-0.006	0.001	0.110
CC	G+C	-0.524	-0.008	0.032
CC	length	0.319	0.004	0.011
GG	genes	-0.300	-0.004	0.026
GG	CDS	-0.612	-0.007	0.042
GG	enhancer	-0.008	-0.019	0.056
GG	G+C	-0.522	0.015	0.032
GG	length	0.319	0.002	0.015
GC	genes	0.181	-0.017	0.012
GC	CDS	0.274	-0.016	0.022
GC	enhancer	0.114	0.017	0.070
GC	G+C	0.468	-0.002	0.030
GC	length	-0.172	0.005	0.011
CG	genes	0.243	0.000	0.016
CG	CDS	0.620	0.004	0.035
CG	enhancer	-0.058	0.006	0.076
CG	G+C	0.236	0.004	0.033
CG	length	-0.347	-0.002	0.015

Table S4: Relative DNA property changes induced by dinucleotide contents: mean values of relative changes (change divided by original value) of physical/structural DNA properties for each dinucleotide (see 2.7 for details on calculations).

id	property name	AA	TT	AT	TA	AC	GT	CA	TG	AG	TC	GA	CT	CC	GG	GC	CG
79	Adenine content (79)	0.23 ± 0.03	-0.09 ± 0.03	0.05 ± 0.0	0.04 ± 0.01	0.04 ± 0.02	-0.05 ± 0.01	0.05 ± 0.02	-0.07 ± 0.01	0.05 ± 0.02	-0.06 ± 0.01	0.05 ± 0.02	-0.06 ± 0.01	-0.05 ± 0.02	-0.05 ± 0.02	-0.05 ± 0.02	-0.03 ± 0.03
4	Bend (4)	-0.0 ± 0.0	-0.0 ± 0.0	-0.01 ± 0.01	0.07 ± 0.02	-0.0 ± 0.0	-0.0 ± 0.0	0.01 ± 0.0	0.01 ± 0.0	-0.02 ± 0.0	-0.01 ± 0.0	-0.01 ± 0.0	-0.02 ± 0.0	-0.02 ± 0.0	-0.02 ± 0.0	-0.0 ± 0.0	-0.0 ± 0.0
21	Clash Strength (21)	-0.04 ± 0.01	-0.04 ± 0.01	0.05 ± 0.02	-0.06 ± 0.03	-0.01 ± 0.0	-0.01 ± 0.0	-0.02 ± 0.0	-0.02 ± 0.0	0.09 ± 0.01	-0.06 ± 0.01	-0.06 ± 0.01	0.09 ± 0.01	0.03 ± 0.01	0.03 ± 0.01	-0.04 ± 0.02	0.04 ± 0.03
81	Cytosine content (81)	-0.09 ± 0.03	-0.09 ± 0.03	-0.08 ± 0.03	-0.06 ± 0.02	0.07 ± 0.02	-0.05 ± 0.01	0.1 ± 0.03	-0.07 ± 0.01	-0.06 ± 0.01	0.09 ± 0.02	-0.06 ± 0.01	0.09 ± 0.02	0.18 ± 0.03	-0.05 ± 0.02	0.06 ± 0.01	0.04 ± 0.02
100	Direction (100)	-2.39 ± 4.5	2.2 ± 4.17	-0.08 ± 0.03	-0.06 ± 0.03	0.98 ± 0.96	-1.08 ± 0.93	-0.66 ± 0.5	0.52 ± 0.49	-0.04 ± 0.02	-1.05 ± 0.79	0.93 ± 0.85	-0.08 ± 0.02	-0.4 ± 0.16	0.3 ± 0.16	0.96 ± 0.25	-0.03 ± 0.03
123	Enthalpy (123)	-0.01 ± 0.0	-0.01 ± 0.0	-0.01 ± 0.0	-0.01 ± 0.0	0.0 ± 0.0	0.0 ± 0.0	0.0 ± 0.0	0.0 ± 0.0	-0.0 ± 0.0	0.0 ± 0.0	0.0 ± 0.0	-0.0 ± 0.0	-0.0 ± 0.0	-0.0 ± 0.0	0.01 ± 0.0	0.01 ± 0.01

22	Enthalpy (22)	-0.0 ± 0.0	-0.0 ± 0.0	-0.03 ± 0.01	-0.01 ± 0.0	0.01 ± 0.0	0.01 ± 0.0	-0.0 ± 0.0	-0.0 ± 0.0	-0.01 ± 0.0	0.0 ± 0.0	0.0 ± 0.0	-0.01 ± 0.0	0.01 ± 0.0	0.01 ± 0.0	0.01 ± 0.0	0.01 ± 0.01
110	Enthalpy (RNA) (110)	-0.02 ± 0.0	-0.02 ± 0.0	-0.03 ± 0.01	-0.01 ± 0.0	0.01 ± 0.0	0.01 ± 0.0	0.01 ± 0.0	-0.01 ± 0.0	-0.01 ± 0.0	0.01 ± 0.0	0.03 ± 0.0	-0.01 ± 0.0	0.02 ± 0.0	0.02 ± 0.0	0.03 ± 0.01	-0.0 ± 0.01
114	Enthalpy (RNA) (114)	-0.03 ± 0.01	-0.03 ± 0.01	-0.01 ± 0.0	-0.02 ± 0.0	0.01 ± 0.0	0.01 ± 0.0	0.0 ± 0.0	0.0 ± 0.0	0.0 ± 0.0	0.01 ± 0.0	0.01 ± 0.0	0.0 ± 0.0	0.01 ± 0.0	0.01 ± 0.0	0.02 ± 0.01	-0.0 ± 0.0
124	Entropy (124)	-0.0 ± 0.0	-0.0 ± 0.0	-0.01 ± 0.0	-0.0 ± 0.0	0.0 ± 0.0	0.0 ± 0.0	0.0 ± 0.0	0.0 ± 0.0	-0.0 ± 0.0	0.0 ± 0.0	0.0 ± 0.0	-0.0 ± 0.0	-0.0 ± 0.0	-0.0 ± 0.0	0.01 ± 0.0	0.01 ± 0.01
23	Entropy (23)	0.0 ± 0.0	0.0 ± 0.0	-0.02 ± 0.01	-0.01 ± 0.0	0.01 ± 0.0	0.01 ± 0.0	-0.0 ± 0.0	-0.0 ± 0.0	-0.02 ± 0.0	0.0 ± 0.0	0.0 ± 0.0	-0.02 ± 0.0	0.01 ± 0.0	0.01 ± 0.0	0.01 ± 0.0	0.01 ± 0.01
111	Entropy (RNA) (111)	-0.02 ± 0.0	-0.02 ± 0.0	-0.03 ± 0.01	-0.0 ± 0.0	0.01 ± 0.0	0.01 ± 0.0	0.01 ± 0.0	-0.01 ± 0.0	-0.01 ± 0.0	0.01 ± 0.0	0.03 ± 0.0	-0.01 ± 0.0	0.01 ± 0.0	0.01 ± 0.0	0.02 ± 0.01	-0.01 ± 0.01
115	Entropy (RNA) (115)	-0.03 ± 0.01	-0.03 ± 0.01	-0.0 ± 0.0	-0.01 ± 0.0	0.0 ± 0.0	0.0 ± 0.0	-0.0 ± 0.0	-0.0 ± 0.0	0.0 ± 0.0	0.01 ± 0.0	0.01 ± 0.0	0.0 ± 0.0	0.01 ± 0.0	0.01 ± 0.0	0.02 ± 0.01	-0.0 ± 0.0
122	Flexibility_shift (122)	-0.01 ± 0.0	-0.01 ± 0.0	-0.06 ± 0.02	-0.02 ± 0.01	0.03 ± 0.0	0.03 ± 0.0	-0.02 ± 0.0	-0.02 ± 0.0	0.03 ± 0.0	-0.01 ± 0.0	-0.01 ± 0.0	0.03 ± 0.0	-0.0 ± 0.0	-0.0 ± 0.0	-0.03 ± 0.02	0.03 ± 0.03
121	Flexibility_slide (121)	0.06 ± 0.01	0.06 ± 0.01	0.03 ± 0.0	-0.01 ± 0.01	0.01 ± 0.0	0.01 ± 0.0	-0.06 ± 0.01	-0.06 ± 0.01	-0.01 ± 0.0	0.01 ± 0.01	0.01 ± 0.01	-0.01 ± 0.0	-0.01 ± 0.0	-0.01 ± 0.0	-0.02 ± 0.01	-0.02 ± 0.01
125	Free energy (125)	-0.02 ± 0.0	-0.02 ± 0.0	-0.02 ± 0.0	-0.03 ± 0.01	0.0 ± 0.0	0.0 ± 0.0	0.01 ± 0.0	0.01 ± 0.0	-0.0 ± 0.0	-0.0 ± 0.0	-0.0 ± 0.0	-0.0 ± 0.0	0.02 ± 0.0	0.02 ± 0.0	0.03 ± 0.01	0.02 ± 0.01
34	Free energy (34)	-0.04 ± 0.01	-0.04 ± 0.01	-0.03 ± 0.01	-0.02 ± 0.01	0.01 ± 0.0	0.01 ± 0.0	0.0 ± 0.01	0.0 ± 0.01	0.0 ± 0.0	0.0 ± 0.0	0.0 ± 0.0	0.0 ± 0.0	0.02 ± 0.0	0.02 ± 0.0	0.03 ± 0.01	0.02 ± 0.01
35	Free energy (35)	0.0 ± 0.01	0.0 ± 0.01	-0.02 ± 0.0	-0.03 ± 0.01	-0.02 ± 0.0	-0.02 ± 0.0	0.01 ± 0.0	0.01 ± 0.0	-0.01 ± 0.0	-0.01 ± 0.0	-0.01 ± 0.0	-0.01 ± 0.0	0.03 ± 0.01	0.03 ± 0.01	0.03 ± 0.01	0.03 ± 0.02
36	Free energy (36)	-0.02 ± 0.0	-0.02 ± 0.0	-0.03 ± 0.01	-0.02 ± 0.01	0.01 ± 0.0	0.01 ± 0.0	0.01 ± 0.0	0.01 ± 0.0	-0.0 ± 0.0	-0.0 ± 0.0	0.0 ± 0.0	-0.0 ± 0.0	0.02 ± 0.0	0.02 ± 0.0	0.03 ± 0.01	0.02 ± 0.01
38	Free energy (38)	-0.04 ± 0.01	-0.04 ± 0.01	-0.05 ± 0.01	-0.05 ± 0.02	0.01 ± 0.01	0.01 ± 0.01	0.01 ± 0.01	0.01 ± 0.01	0.0 ± 0.01	0.01 ± 0.01	0.01 ± 0.01	0.0 ± 0.01	0.02 ± 0.0	0.02 ± 0.0	0.04 ± 0.01	0.03 ± 0.02
72	Free energy (72)	-0.02 ± 0.0	-0.02 ± 0.0	-0.03 ± 0.01	-0.03 ± 0.01	-0.0 ± 0.0	-0.0 ± 0.0	0.01 ± 0.0	0.01 ± 0.0	-0.0 ± 0.0	-0.0 ± 0.0	-0.0 ± 0.0	-0.0 ± 0.0	0.02 ± 0.0	0.02 ± 0.0	0.02 ± 0.01	0.03 ± 0.02
73	Free energy (73)	-0.02 ± 0.0	-0.02 ± 0.0	-0.02 ± 0.0	-0.03 ± 0.01	0.0 ± 0.0	0.0 ± 0.0	0.01 ± 0.0	0.01 ± 0.0	-0.0 ± 0.0	-0.0 ± 0.0	-0.0 ± 0.0	-0.0 ± 0.0	0.02 ± 0.0	0.02 ± 0.0	0.03 ± 0.01	0.02 ± 0.01
74	Free energy (74)	-0.02 ± 0.0	-0.02 ± 0.0	-0.03 ± 0.01	-0.02 ± 0.01	0.0 ± 0.0	0.0 ± 0.0	0.02 ± 0.0	0.02 ± 0.0	-0.01 ± 0.0	0.0 ± 0.0	0.0 ± 0.0	-0.01 ± 0.0	0.01 ± 0.0	0.01 ± 0.0	0.03 ± 0.01	0.02 ± 0.01
75	Free energy (75)	-0.02 ± 0.0	-0.02 ± 0.0	-0.03 ± 0.0	-0.03 ± 0.01	-0.0 ± 0.0	-0.0 ± 0.0	0.01 ± 0.0	0.01 ± 0.0	0.0 ± 0.0	0.0 ± 0.0	0.0 ± 0.0	0.0 ± 0.0	0.02 ± 0.0	0.02 ± 0.0	0.02 ± 0.0	0.01 ± 0.01
112	Free energy (RNA) (112)	-0.04 ± 0.01	-0.04 ± 0.01	-0.04 ± 0.01	-0.02 ± 0.01	0.01 ± 0.0	0.01 ± 0.0	0.0 ± 0.01	-0.0 ± 0.01	-0.0 ± 0.0	0.01 ± 0.01	0.02 ± 0.01	-0.0 ± 0.0	0.03 ± 0.0	0.03 ± 0.0	0.04 ± 0.01	0.0 ± 0.0
113	Free energy (RNA) (113)	-0.05 ± 0.01	-0.05 ± 0.01	-0.03 ± 0.01	-0.02 ± 0.0	0.01 ± 0.0	0.01 ± 0.0	0.0 ± 0.01	0.0 ± 0.01	0.0 ± 0.0	0.01 ± 0.01	0.01 ± 0.01	0.0 ± 0.0	0.03 ± 0.0	0.03 ± 0.0	0.03 ± 0.01	0.0 ± 0.0
76	GC content (76)	-0.09 ± 0.03	-0.09 ± 0.03	-0.08 ± 0.03	-0.06 ± 0.02	0.01 ± 0.01	0.01 ± 0.01	0.01 ± 0.02	0.01 ± 0.02	0.01 ± 0.01	0.01 ± 0.01	0.01 ± 0.01	0.01 ± 0.01	0.06 ± 0.01	0.06 ± 0.01	0.06 ± 0.01	0.04 ± 0.02
80	Guanine content (80)	-0.09 ± 0.03	-0.09 ± 0.03	-0.08 ± 0.03	-0.06 ± 0.02	-0.05 ± 0.01	0.07 ± 0.02	-0.07 ± 0.01	0.1 ± 0.03	0.09 ± 0.02	-0.06 ± 0.01	0.09 ± 0.02	-0.06 ± 0.01	-0.05 ± 0.02	0.18 ± 0.03	0.06 ± 0.01	0.04 ± 0.02
29	Hydrophilicity (RNA) (29)	-0.08 ± 0.02	0.12 ± 0.04	-0.04 ± 0.01	-0.02 ± 0.01	-0.03 ± 0.0	-0.0 ± 0.0	-0.02 ± 0.0	0.02 ± 0.0	-0.05 ± 0.01	0.07 ± 0.01	-0.04 ± 0.01	0.07 ± 0.01	0.05 ± 0.01	-0.03 ± 0.01	-0.01 ± 0.0	0.0 ± 0.0
31	Hydrophilicity (RNA) (31)	-0.08 ± 0.02	0.07 ± 0.02	-0.03 ± 0.01	-0.01 ± 0.0	-0.02 ± 0.0	0.0 ± 0.0	-0.01 ± 0.0	0.02 ± 0.0	-0.04 ± 0.01	0.05 ± 0.0	-0.04 ± 0.0	0.06 ± 0.01	0.04 ± 0.01	-0.02 ± 0.01	-0.0 ± 0.0	0.01 ± 0.01
6	Inclination (6)	-17.39 ± 9309.53	18.01 ± 9304.96	-0.07 ± 0.64	-0.06 ± 0.53	-2.14 ± 411.8	2.06 ± 411.5	61.23 ± 7112.29	-61.16 ± 7100.99	-50.21 ± 4606.19	10.32 ± 1411.37	-10.27 ± 1409.15	50.33 ± 4608.17	-65.5 ± 4882.08	65.1 ± 4872.58	-0.03 ± 0.45	-0.02 ± 0.34
78	Keto (GT) content (78)	-0.09 ± 0.03	0.12 ± 0.03	0.01 ± 0.0	0.01 ± 0.0	-0.05 ± 0.01	0.07 ± 0.01	-0.07 ± 0.01	0.01 ± 0.0	-0.06 ± 0.01	0.01 ± 0.0	0.01 ± 0.0	0.01 ± 0.0	-0.05 ± 0.02	0.06 ± 0.02	0.01 ± 0.0	0.0 ± 0.0
8	Major Groove Depth (8)	0.0 ± 0.0	0.0 ± 0.0	-0.0 ± 0.0	0.0 ± 0.0	0.0 ± 0.0	0.0 ± 0.0	-0.0 ± 0.0	-0.0 ± 0.0	-0.0 ± 0.0	-0.0 ± 0.0	-0.0 ± 0.0	-0.0 ± 0.0	-0.0 ± 0.0	-0.0 ± 0.0	-0.0 ± 0.0	-0.0 ± 0.0
10	Major Groove Distance (10)	-0.0 ± 0.0	-0.0 ± 0.0	-0.01 ± 0.0	0.01 ± 0.0	-0.01 ± 0.0	-0.01 ± 0.0	0.01 ± 0.0	0.01 ± 0.0	-0.0 ± 0.0	0.0 ± 0.0	0.0 ± 0.0	-0.0 ± 0.0	-0.0 ± 0.0	-0.0 ± 0.0	-0.01 ± 0.0	0.0 ± 0.0
9	Major Groove Size (9)	0.0 ± 0.0	0.0 ± 0.0	0.01 ± 0.0	-0.01 ± 0.0	0.0 ± 0.0	0.0 ± 0.0	0.0 ± 0.0	0.0 ± 0.0	0.01 ± 0.0	-0.01 ± 0.0	-0.01 ± 0.0	0.01 ± 0.0	0.0 ± 0.0	0.0 ± 0.0	-0.01 ± 0.0	0.01 ± 0.01
7	Major Groove Width (7)	-0.01 ± 0.0	-0.01 ± 0.0	-0.0 ± 0.0	-0.0 ± 0.0	-0.0 ± 0.0	-0.0 ± 0.0	0.0 ± 0.0	0.0 ± 0.0	0.0 ± 0.0	0.0 ± 0.0	0.0 ± 0.0	0.0 ± 0.0	0.01 ± 0.0	0.01 ± 0.0	0.0 ± 0.0	0.0 ± 0.0
108	Melting Temperature (108)	-0.0 ± 0.0	-0.0 ± 0.0	-0.0 ± 0.0	-0.01 ± 0.0	0.0 ± 0.0	0.0 ± 0.0	-0.0 ± 0.0	-0.0 ± 0.0	-0.0 ± 0.0	0.0 ± 0.0	0.0 ± 0.0	-0.0 ± 0.0	0.0 ± 0.0	0.0 ± 0.0	0.01 ± 0.0	-0.0 ± 0.0

16	Melting Temperature (16)	-0.02 ± 0.0	-0.02 ± 0.0	-0.01 ± 0.0	-0.03 ± 0.01	0.02 ± 0.0	0.02 ± 0.0	-0.02 ± 0.0	-0.02 ± 0.0	-0.01 ± 0.0	0.01 ± 0.0	0.01 ± 0.0	-0.01 ± 0.0	0.01 ± 0.0	0.01 ± 0.0	0.04 ± 0.01	-0.0 ± 0.0
12	Minor Groove Depth (12)	0.0 ± 0.0	0.0 ± 0.0	-0.0 ± 0.0	0.0 ± 0.0	-0.0 ± 0.0	-0.0 ± 0.0	0.0 ± 0.0	0.0 ± 0.0	-0.0 ± 0.0	0.0 ± 0.0	0.0 ± 0.0	-0.0 ± 0.0	-0.0 ± 0.0	-0.0 ± 0.0	-0.0 ± 0.0	0.0 ± 0.0
14	Minor Groove Distance (14)	-0.01 ± 0.0	-0.01 ± 0.0	0.02 ± 0.01	-0.01 ± 0.0	0.02 ± 0.0	0.02 ± 0.0	-0.0 ± 0.0	-0.0 ± 0.0	-0.01 ± 0.0	-0.01 ± 0.0	-0.01 ± 0.0	-0.01 ± 0.0	-0.01 ± 0.0	-0.01 ± 0.0	0.01 ± 0.01	-0.0 ± 0.0
13	Minor Groove Size (13)	-0.01 ± 0.0	-0.01 ± 0.0	-0.0 ± 0.0	-0.01 ± 0.0	-0.0 ± 0.0	-0.0 ± 0.0	0.01 ± 0.0	0.01 ± 0.0	0.01 ± 0.0	-0.01 ± 0.0	-0.01 ± 0.0	0.01 ± 0.0	0.01 ± 0.0	0.01 ± 0.0	-0.0 ± 0.0	0.01 ± 0.01
11	Minor Groove Width (11)	0.0 ± 0.0	0.0 ± 0.0	0.0 ± 0.0	0.01 ± 0.0	0.01 ± 0.0	0.01 ± 0.0	-0.01 ± 0.0	-0.01 ± 0.0	-0.0 ± 0.0	-0.01 ± 0.0	-0.01 ± 0.0	-0.0 ± 0.0	-0.01 ± 0.0	-0.01 ± 0.0	-0.0 ± 0.0	-0.0 ± 0.0
18	Mobility to bend towards major groove (18)	0.01 ± 0.0	0.0 ± 0.0	0.0 ± 0.0	0.0 ± 0.0	0.0 ± 0.0	-0.0 ± 0.0	0.0 ± 0.0	-0.0 ± 0.0	0.0 ± 0.0	-0.0 ± 0.0	0.0 ± 0.0	-0.0 ± 0.0	-0.0 ± 0.0	-0.0 ± 0.0	-0.0 ± 0.0	-0.0 ± 0.0
19	Mobility to bend towards minor groove (19)	-0.01 ± 0.0	-0.01 ± 0.0	-0.01 ± 0.0	-0.0 ± 0.0	-0.0 ± 0.0	-0.0 ± 0.0	0.0 ± 0.0	0.01 ± 0.0	-0.0 ± 0.0	0.0 ± 0.0	-0.0 ± 0.0	0.0 ± 0.0	0.01 ± 0.0	0.01 ± 0.0	0.0 ± 0.0	0.0 ± 0.0
15	Persistance Length (15)	-0.03 ± 0.01	-0.03 ± 0.01	-0.05 ± 0.01	-0.04 ± 0.01	0.0 ± 0.01	0.0 ± 0.01	0.0 ± 0.01	0.0 ± 0.01	0.0 ± 0.01	0.0 ± 0.01	0.0 ± 0.01	0.0 ± 0.01	0.06 ± 0.01	0.06 ± 0.01	0.02 ± 0.0	0.01 ± 0.01
17	Probability contacting nucleosome core (17)	0.05 ± 0.01	0.05 ± 0.01	-0.03 ± 0.01	-0.03 ± 0.01	-0.01 ± 0.0	-0.01 ± 0.0	0.02 ± 0.0	0.02 ± 0.0	0.01 ± 0.0	-0.0 ± 0.0	-0.0 ± 0.0	0.02 ± 0.0	-0.01 ± 0.0	-0.01 ± 0.0	-0.03 ± 0.01	-0.03 ± 0.03
20	Propeller Twist (20)	0.03 ± 0.01	0.03 ± 0.01	0.02 ± 0.0	-0.01 ± 0.01	-0.03 ± 0.0	-0.03 ± 0.0	-0.02 ± 0.0	-0.02 ± 0.0	0.01 ± 0.0	0.01 ± 0.0	0.01 ± 0.0	0.01 ± 0.0	0.0 ± 0.0	0.0 ± 0.0	-0.0 ± 0.0	-0.0 ± 0.0
77	Purine (AG) content (77)	0.09 ± 0.03	-0.09 ± 0.03	0.0 ± 0.0	0.0 ± 0.0	0.0 ± 0.0	0.0 ± 0.0	0.0 ± 0.0	0.0 ± 0.0	0.06 ± 0.01	-0.06 ± 0.01	0.06 ± 0.01	-0.06 ± 0.01	-0.05 ± 0.02	0.05 ± 0.02	0.0 ± 0.0	0.0 ± 0.0
3	Rise (3)	-0.01 ± 0.0	-0.01 ± 0.0	0.01 ± 0.0	-0.0 ± 0.0	-0.0 ± 0.0	-0.0 ± 0.0	-0.01 ± 0.0	-0.01 ± 0.0	0.0 ± 0.0	-0.0 ± 0.0	-0.0 ± 0.0	0.0 ± 0.0	0.01 ± 0.0	0.01 ± 0.0	0.0 ± 0.0	0.0 ± 0.0
66	Rise (66)	-0.0 ± 0.0	-0.0 ± 0.0	-0.0 ± 0.0	0.0 ± 0.0	-0.0 ± 0.0	-0.0 ± 0.0	-0.0 ± 0.0	-0.0 ± 0.0	-0.0 ± 0.0	0.0 ± 0.0	0.0 ± 0.0	-0.0 ± 0.0	0.0 ± 0.0	0.0 ± 0.0	0.0 ± 0.0	-0.0 ± 0.0
97	Rise (97)	-0.0 ± 0.0	-0.0 ± 0.0	-0.0 ± 0.0	0.0 ± 0.0	-0.0 ± 0.0	-0.0 ± 0.0	0.0 ± 0.0	0.0 ± 0.0	-0.0 ± 0.0	-0.0 ± 0.0	-0.0 ± 0.0	-0.0 ± 0.0	0.0 ± 0.0	0.0 ± 0.0	0.0 ± 0.0	0.0 ± 0.0
32	Rise (DNA-protein complex) (32)	-0.0 ± 0.0	-0.0 ± 0.0	-0.0 ± 0.0	0.0 ± 0.0	0.0 ± 0.0	0.0 ± 0.0	0.0 ± 0.0	0.0 ± 0.0	0.0 ± 0.0	0.0 ± 0.0	0.0 ± 0.0	0.0 ± 0.0	0.0 ± 0.0	0.0 ± 0.0	0.0 ± 0.0	0.0 ± 0.0
87	Rise (DNA-protein complex) (87)	-0.0 ± 0.0	-0.0 ± 0.0	-0.0 ± 0.0	0.0 ± 0.0	0.0 ± 0.0	0.0 ± 0.0	-0.0 ± 0.0	-0.0 ± 0.0	-0.0 ± 0.0	0.0 ± 0.0	0.0 ± 0.0	-0.0 ± 0.0	0.0 ± 0.0	0.0 ± 0.0	0.0 ± 0.0	0.0 ± 0.0
102	Rise (RNA) (102)	-0.0 ± 0.0	-0.0 ± 0.0	-0.0 ± 0.0	0.0 ± 0.0	-0.0 ± 0.0	-0.0 ± 0.0	-0.0 ± 0.0	-0.0 ± 0.0	0.0 ± 0.0	0.0 ± 0.0	0.0 ± 0.0	0.0 ± 0.0	0.0 ± 0.0	0.0 ± 0.0	-0.0 ± 0.0	0.0 ± 0.0
107	Rise stiffness (107)	-0.0 ± 0.0	-0.0 ± 0.0	0.01 ± 0.0	-0.01 ± 0.0	0.01 ± 0.0	0.01 ± 0.0	-0.01 ± 0.0	-0.01 ± 0.0	-0.01 ± 0.0	0.01 ± 0.0	0.01 ± 0.0	-0.01 ± 0.0	0.0 ± 0.0	0.0 ± 0.0	0.01 ± 0.0	-0.01 ± 0.01
47	Rise_rise (47)	-0.0 ± 0.0	-0.0 ± 0.0	0.03 ± 0.01	-0.02 ± 0.01	0.02 ± 0.0	0.02 ± 0.0	-0.01 ± 0.0	-0.01 ± 0.0	-0.01 ± 0.0	0.01 ± 0.0	0.01 ± 0.0	-0.01 ± 0.0	-0.0 ± 0.0	-0.0 ± 0.0	0.02 ± 0.01	-0.01 ± 0.01
116	Roll (116)	1.77 ± 96.97	1.67 ± 96.12	1.7 ± 94.67	-1.65 ± 83.64	0.35 ± 24.89	0.31 ± 23.95	-1.08 ± 79.98	-0.96 ± 78.4	-0.12 ± 9.07	-0.27 ± 18.34	-0.25 ± 17.92	-0.13 ± 9.22	-0.15 ± 10.2	-0.13 ± 9.78	0.4 ± 33.78	-0.47 ± 23.91
119	Roll (119)	0.01 ± 0.01	0.01 ± 0.01	-0.38 ± 0.14	0.19 ± 0.09	-0.1 ± 0.01	-0.1 ± 0.01	0.17 ± 0.02	0.17 ± 0.02	-0.05 ± 0.01	0.08 ± 0.01	0.08 ± 0.01	-0.05 ± 0.01	-0.02 ± 0.01	-0.02 ± 0.01	-0.06 ± 0.03	0.06 ± 0.05
63	Roll (63)	0.03 ± 0.03	0.03 ± 0.03	-0.17 ± 0.07	-0.1 ± 0.05	0.01 ± 0.01	0.01 ± 0.01	-0.14 ± 0.02	-0.14 ± 0.02	0.24 ± 0.03	-0.04 ± 0.01	-0.04 ± 0.01	0.24 ± 0.03	0.12 ± 0.02	0.12 ± 0.02	-0.35 ± 0.14	0.15 ± 0.11
90	Roll (90)	-0.06 ± 0.01	-0.06 ± 0.01	-0.12 ± 0.04	0.07 ± 0.04	-0.04 ± 0.01	-0.04 ± 0.01	-0.01 ± 0.01	-0.01 ± 0.01	0.07 ± 0.01	-0.07 ± 0.01	-0.07 ± 0.01	0.07 ± 0.01	0.2 ± 0.04	0.2 ± 0.03	-0.32 ± 0.13	0.13 ± 0.1
94	Roll (94)	-0.07 ± 0.02	-0.07 ± 0.02	-0.14 ± 0.05	0.1 ± 0.06	-0.03 ± 0.01	-0.03 ± 0.01	-0.04 ± 0.01	-0.04 ± 0.01	0.19 ± 0.02	-0.14 ± 0.02	-0.14 ± 0.02	0.19 ± 0.02	0.22 ± 0.05	0.22 ± 0.04	-0.33 ± 0.16	0.06 ± 0.05
25	Roll (DNA-protein complex) (25)	-0.06 ± 0.02	-0.06 ± 0.02	-0.08 ± 0.03	0.01 ± 0.01	-0.06 ± 0.01	-0.06 ± 0.01	0.1 ± 0.01	0.1 ± 0.01	0.07 ± 0.01	-0.0 ± 0.0	-0.0 ± 0.0	0.07 ± 0.01	0.01 ± 0.0	0.01 ± 0.0	-0.09 ± 0.04	0.05 ± 0.04
84	Roll (DNA-protein complex) (84)	-0.07 ± 0.02	-0.07 ± 0.02	-0.04 ± 0.01	0.02 ± 0.02	-0.04 ± 0.0	-0.04 ± 0.0	0.06 ± 0.01	0.06 ± 0.01	0.05 ± 0.01	-0.01 ± 0.0	-0.01 ± 0.0	0.05 ± 0.01	0.02 ± 0.0	0.02 ± 0.0	-0.04 ± 0.02	0.04 ± 0.03
104	Roll (RNA) (104)	-0.02 ± 0.0	-0.02 ± 0.0	-0.01 ± 0.0	0.02 ± 0.01	-0.02 ± 0.0	-0.02 ± 0.0	0.01 ± 0.0	0.01 ± 0.0	0.0 ± 0.0	0.01 ± 0.0	0.01 ± 0.0	0.0 ± 0.0	0.0 ± 0.0	0.02 ± 0.01	-0.01 ± 0.01	0.01 ± 0.01
69	Roll stiffness (69)	0.0 ± 0.0	0.0 ± 0.0	0.01 ± 0.0	-0.01 ± 0.0	0.01 ± 0.0	0.01 ± 0.0	-0.01 ± 0.0	-0.01 ± 0.0	-0.0 ± 0.0	0.0 ± 0.0	0.0 ± 0.0	-0.0 ± 0.0	-0.0 ± 0.0	-0.0 ± 0.0	0.02 ± 0.01	-0.01 ± 0.01
59	Roll_rise (59)	2.84 ± 60.22	2.84 ± 60.1	-3.95 ± 86.01	1.39 ± 30.72	-2.95 ± 66.88	-2.94 ± 66.92	3.05 ± 70.35	3.04 ± 70.46	0.06 ± 2.61	0.4 ± 10.48	0.4 ± 10.51	0.06 ± 2.6	-0.09 ± 1.52	-0.09 ± 1.58	-2.83 ± 76.88	0.3 ± 10.55
41	Roll_roll (41)	0.01 ± 0.0	0.01 ± 0.0	0.02 ± 0.01	-0.02 ± 0.01	0.01 ± 0.0	0.01 ± 0.0	-0.01 ± 0.0	-0.01 ± 0.0	0.0 ± 0.0	-0.0 ± 0.0	-0.0 ± 0.0	0.0 ± 0.0	0.0 ± 0.0	0.0 ± 0.0	0.01 ± 0.01	-0.01 ± 0.01
57	Roll_shift (57)	0.31 ± 0.06	0.31 ± 0.06	0.11 ± 0.02	-0.23 ± 0.07	0.16 ± 0.06	0.16 ± 0.06	-0.12 ± 0.03	-0.12 ± 0.03	-0.33 ± 0.12	0.14 ± 0.05	0.14 ± 0.05	-0.33 ± 0.12	-0.12 ± 0.07	-0.12 ± 0.07	-0.07 ± 0.04	0.08 ± 0.09

58	Roll_slide (58)	0.07 ± 0.01	0.07 ± 0.01	0.23 ± 0.03	-0.03 ± 0.02	-0.05 ± 0.0	-0.05 ± 0.0	-0.12 ± 0.02	-0.12 ± 0.02	0.09 ± 0.03	-0.06 ± 0.01	-0.06 ± 0.01	0.09 ± 0.03	-0.02 ± 0.0	-0.02 ± 0.0	-0.08 ± 0.04	0.03 ± 0.04
64	Shift (64)	2.49 ± 20.28	2.5 ± 20.26	-4.57 ± 38.19	5.12 ± 43.84	-1.83 ± 16.61	-1.83 ± 16.59	-0.86 ± 7.69	-0.86 ± 7.68	-2.1 ± 19.24	-0.1 ± 0.76	-0.1 ± 0.76	-2.1 ± 19.26	-1.47 ± 13.58	-1.47 ± 13.66	7.47 ± 66.45	-0.98 ± 8.87
95	Shift (95)	-0.12 ± 0.77	-0.12 ± 0.8	-0.1 ± 0.66	-0.08 ± 0.65	-1.23 ± 7.54	-1.23 ± 7.84	-0.77 ± 4.22	-0.78 ± 4.42	2.28 ± 10.79	-0.07 ± 0.3	-0.07 ± 0.3	2.29 ± 10.89	-0.06 ± 0.24	-0.06 ± 0.24	-0.06 ± 0.23	-0.05 ± 0.22
30	Shift (DNA-protein complex) (30)	0.37 ± 0.33	0.37 ± 0.33	-0.07 ± 0.03	-0.05 ± 0.03	-0.3 ± 0.59	-0.3 ± 0.6	-0.06 ± 0.05	-0.06 ± 0.05	-0.67 ± 1.18	0.88 ± 1.32	0.87 ± 1.32	-0.67 ± 1.19	-0.04 ± 0.06	-0.04 ± 0.06	-0.04 ± 0.08	-0.02 ± 0.06
85	Shift (DNA-protein complex) (85)	0.71 ± 24.38	0.71 ± 24.41	0.05 ± 2.17	0.04 ± 1.6	-1.67 ± 56.97	-1.65 ± 57.12	-1.27 ± 45.92	-1.26 ± 46.11	-1.23 ± 43.14	4.89 ± 154.95	4.84 ± 155.14	-1.25 ± 43.14	-0.53 ± 19.26	-0.52 ± 19.25	0.07 ± 2.96	0.08 ± 2.98
24	Shift (RNA) (24)	-0.32 ± 0.49	-0.32 ± 0.49	-0.22 ± 0.33	-0.11 ± 0.11	0.29 ± 0.33	0.29 ± 0.32	0.14 ± 0.2	0.14 ± 0.2	-0.15 ± 0.08	0.05 ± 0.11	0.05 ± 0.11	-0.15 ± 0.08	-0.08 ± 0.03	-0.08 ± 0.03	0.04 ± 0.04	0.21 ± 0.14
68	Shift stiffness (68)	0.03 ± 0.01	0.03 ± 0.01	-0.02 ± 0.0	-0.03 ± 0.01	0.0 ± 0.0	0.0 ± 0.0	-0.01 ± 0.0	-0.01 ± 0.0	0.01 ± 0.0	0.0 ± 0.0	0.0 ± 0.0	0.01 ± 0.0	0.01 ± 0.0	0.01 ± 0.0	-0.0 ± 0.0	-0.01 ± 0.0
49	Shift_rise (49)	0.29 ± 0.08	0.29 ± 0.08	-0.11 ± 0.03	-0.1 ± 0.04	-0.06 ± 0.01	-0.06 ± 0.01	-0.15 ± 0.02	-0.15 ± 0.02	-0.1 ± 0.02	-0.02 ± 0.01	-0.02 ± 0.01	-0.1 ± 0.02	0.18 ± 0.05	0.18 ± 0.05	0.09 ± 0.04	-0.06 ± 0.06
45	Shift_shift (45)	0.02 ± 0.01	0.02 ± 0.01	-0.02 ± 0.01	-0.0 ± 0.0	-0.01 ± 0.0	-0.01 ± 0.0	-0.0 ± 0.0	-0.0 ± 0.0	0.0 ± 0.0	-0.01 ± 0.0	-0.01 ± 0.0	0.0 ± 0.0	0.01 ± 0.0	0.01 ± 0.0	0.0 ± 0.0	-0.01 ± 0.01
48	Shift_slide (48)	-2.37 ± 31.07	-2.38 ± 31.11	0.89 ± 13.16	-0.57 ± 7.33	0.89 ± 9.45	0.89 ± 9.47	2.12 ± 21.03	2.13 ± 21.13	0.09 ± 1.34	-0.28 ± 2.29	-0.28 ± 2.3	0.09 ± 1.34	-0.38 ± 2.54	-0.38 ± 2.55	-1.58 ± 9.74	0.52 ± 3.94
65	Slide (65)	-0.15 ± 0.07	-0.15 ± 0.07	-0.26 ± 0.18	0.05 ± 0.08	-0.14 ± 0.02	-0.14 ± 0.02	0.43 ± 0.1	0.43 ± 0.1	0.02 ± 0.02	-0.06 ± 0.01	-0.06 ± 0.01	0.02 ± 0.02	0.01 ± 0.01	0.01 ± 0.01	0.01 ± 0.01	0.05 ± 0.03
91	Slide (91)	-0.1 ± 0.03	-0.1 ± 0.03	-0.16 ± 0.07	0.08 ± 0.06	-0.07 ± 0.01	-0.07 ± 0.01	0.23 ± 0.03	0.23 ± 0.03	0.03 ± 0.01	-0.07 ± 0.01	-0.07 ± 0.01	0.03 ± 0.01	0.04 ± 0.0	0.04 ± 0.0	-0.01 ± 0.01	0.03 ± 0.02
96	Slide (96)	-0.12 ± 0.04	-0.12 ± 0.04	-0.16 ± 0.07	0.1 ± 0.07	-0.08 ± 0.01	-0.08 ± 0.01	0.22 ± 0.03	0.23 ± 0.03	0.01 ± 0.01	-0.06 ± 0.01	-0.06 ± 0.01	0.01 ± 0.01	0.06 ± 0.01	0.06 ± 0.01	0.0 ± 0.01	0.03 ± 0.02
28	Slide (DNA-protein complex) (28)	-0.24 ± 1.15	-0.24 ± 1.17	0.71 ± 4.37	-0.16 ± 0.48	0.44 ± 5.13	0.44 ± 5.13	-0.5 ± 3.4	-0.5 ± 3.41	0.22 ± 1.78	-0.16 ± 1.76	-0.16 ± 1.75	0.22 ± 1.81	0.03 ± 0.48	0.03 ± 0.48	0.18 ± 4.27	-0.39 ± 17.42
86	Slide (DNA-protein complex) (86)	-0.02 ± 0.01	-0.02 ± 0.01	0.39 ± 0.08	-0.1 ± 0.04	0.28 ± 0.06	0.28 ± 0.06	-0.46 ± 0.08	-0.46 ± 0.08	0.1 ± 0.02	-0.13 ± 0.03	-0.13 ± 0.03	0.1 ± 0.02	0.07 ± 0.03	0.07 ± 0.03	0.16 ± 0.1	-0.21 ± 0.22
101	Slide (RNA) (101)	-0.01 ± 0.0	-0.01 ± 0.0	-0.01 ± 0.0	-0.0 ± 0.0	-0.0 ± 0.0	-0.0 ± 0.0	-0.0 ± 0.0	-0.0 ± 0.0	0.0 ± 0.0	0.01 ± 0.0	0.01 ± 0.0	0.0 ± 0.0	0.01 ± 0.0	0.01 ± 0.0	-0.0 ± 0.0	0.01 ± 0.01
67	Slide stiffness (67)	0.0 ± 0.0	0.0 ± 0.0	0.06 ± 0.02	-0.03 ± 0.01	0.02 ± 0.0	0.02 ± 0.0	-0.01 ± 0.0	-0.01 ± 0.0	-0.01 ± 0.0	-0.01 ± 0.0	-0.01 ± 0.0	-0.01 ± 0.0	-0.01 ± 0.0	-0.01 ± 0.0	0.01 ± 0.01	-0.0 ± 0.0
50	Slide_rise (50)	-0.01 ± 0.0	-0.01 ± 0.0	0.05 ± 0.02	-0.03 ± 0.01	0.04 ± 0.0	0.04 ± 0.0	-0.03 ± 0.0	-0.03 ± 0.0	-0.01 ± 0.0	-0.02 ± 0.0	-0.01 ± 0.0	-0.01 ± 0.0	-0.01 ± 0.0	-0.01 ± 0.0	0.03 ± 0.02	-0.0 ± 0.0
46	Slide_slide (46)	0.01 ± 0.0	0.01 ± 0.0	0.02 ± 0.0	-0.01 ± 0.0	0.0 ± 0.0	0.0 ± 0.0	-0.01 ± 0.0	-0.01 ± 0.0	-0.0 ± 0.0	-0.0 ± 0.0	-0.0 ± 0.0	-0.0 ± 0.0	0.01 ± 0.0	0.01 ± 0.0	-0.0 ± 0.0	-0.01 ± 0.01
109	Stacking energy (109)	-0.02 ± 0.0	-0.02 ± 0.0	-0.01 ± 0.0	-0.02 ± 0.01	0.02 ± 0.0	0.02 ± 0.0	-0.01 ± 0.0	-0.01 ± 0.0	-0.01 ± 0.0	0.02 ± 0.0	0.02 ± 0.0	-0.01 ± 0.0	0.0 ± 0.0	0.0 ± 0.0	0.04 ± 0.01	0.0 ± 0.0
2	Stacking energy (2)	0.0 ± 0.0	0.0 ± 0.0	-0.01 ± 0.0	-0.0 ± 0.0	0.0 ± 0.0	0.0 ± 0.0	0.0 ± 0.0	0.0 ± 0.0	-0.0 ± 0.0	-0.0 ± 0.0	-0.0 ± 0.0	-0.0 ± 0.0	-0.01 ± 0.0	-0.01 ± 0.0	0.01 ± 0.0	0.0 ± 0.0
33	Stacking energy (33)	-0.03 ± 0.0	-0.03 ± 0.0	-0.01 ± 0.0	-0.03 ± 0.01	0.02 ± 0.0	0.02 ± 0.0	-0.01 ± 0.0	-0.01 ± 0.0	-0.01 ± 0.0	0.02 ± 0.0	0.02 ± 0.0	-0.01 ± 0.0	0.0 ± 0.0	0.0 ± 0.0	0.04 ± 0.01	0.01 ± 0.0
60	Stacking energy (60)	0.0 ± 0.0	0.0 ± 0.0	-0.0 ± 0.0	0.0 ± 0.0	0.0 ± 0.0	0.0 ± 0.0	0.01 ± 0.0	0.01 ± 0.0	-0.0 ± 0.0	-0.01 ± 0.0	-0.01 ± 0.0	-0.0 ± 0.0	-0.01 ± 0.0	-0.01 ± 0.0	-0.01 ± 0.0	0.0 ± 0.0
106	Stacking energy (RNA) (106)	-0.0 ± 0.0	-0.0 ± 0.0	0.01 ± 0.0	0.01 ± 0.0	-0.0 ± 0.0	-0.0 ± 0.0	0.0 ± 0.0	0.0 ± 0.0	-0.0 ± 0.0	-0.0 ± 0.0	-0.0 ± 0.0	-0.0 ± 0.0	-0.01 ± 0.0	-0.01 ± 0.0	0.01 ± 0.0	0.0 ± 0.0
82	Thymine content (82)	-0.09 ± 0.03	0.23 ± 0.03	0.05 ± 0.0	0.04 ± 0.01	-0.05 ± 0.01	0.04 ± 0.02	-0.07 ± 0.01	0.05 ± 0.02	-0.06 ± 0.01	0.05 ± 0.02	-0.06 ± 0.01	0.05 ± 0.02	-0.05 ± 0.02	-0.05 ± 0.02	-0.05 ± 0.02	-0.03 ± 0.03
117	Tilt (117)	-12.61 ± 560.88	12.57 ± 560.53	-0.06 ± 0.37	-0.05 ± 0.31	-26.17 ± 1498.74	26.33 ± 1495.69	6.11 ± 300.79	-6.22 ± 299.97	-24.29 ± 1111.63	21.57 ± 1108.01	-21.3 ± 1106.24	24.44 ± 1112.79	6.09 ± 324.05	-6.11 ± 323.29	-0.04 ± 0.27	-0.03 ± 0.19
62	Tilt (62)	-0.0 ± 17.77	-0.01 ± 17.27	0.04 ± 32.9	0.06 ± 85.56	-0.23 ± 27.46	-0.23 ± 27.5	-0.01 ± 5.09	-0.01 ± 5.12	0.31 ± 35.09	0.02 ± 6.02	0.02 ± 6.23	0.28 ± 33.96	0.35 ± 27.11	0.37 ± 27.89	0.04 ± 5.88	0.05 ± 5.37
89	Tilt (89)	-0.03 ± 0.0	-0.03 ± 0.0	-0.08 ± 0.03	-0.06 ± 0.03	0.03 ± 0.01	0.03 ± 0.01	-0.14 ± 0.01	-0.14 ± 0.01	0.22 ± 0.01	-0.02 ± 0.01	-0.02 ± 0.01	0.22 ± 0.01	0.05 ± 0.01	0.05 ± 0.01	-0.05 ± 0.03	-0.04 ± 0.03
93	Tilt (93)	-0.02 ± 0.01	-0.02 ± 0.01	-0.07 ± 0.03	-0.06 ± 0.03	-0.04 ± 0.01	-0.04 ± 0.01	-0.13 ± 0.01	-0.13 ± 0.01	0.18 ± 0.02	0.02 ± 0.01	0.02 ± 0.01	0.18 ± 0.02	0.13 ± 0.02	0.13 ± 0.02	-0.05 ± 0.03	-0.04 ± 0.04
27	Tilt (DNA-protein complex) (27)	0.11 ± 0.02	0.11 ± 0.02	-0.07 ± 0.02	-0.06 ± 0.02	-0.03 ± 0.0	-0.06 ± 0.01	-0.04 ± 0.0	-0.04 ± 0.0	0.03 ± 0.01	0.06 ± 0.01	0.06 ± 0.01	0.03 ± 0.01	0.01 ± 0.01	0.01 ± 0.01	-0.05 ± 0.02	-0.03 ± 0.03

83	Tilt (DNA-protein complex) (83)	0.12 ± 0.02	0.12 ± 0.02	-0.07 ± 0.03	-0.06 ± 0.03	-0.04 ± 0.01	-0.04 ± 0.01	-0.12 ± 0.02	-0.12 ± 0.02	0.12 ± 0.02	0.1 ± 0.02	0.1 ± 0.02	0.12 ± 0.02	-0.04 ± 0.01	-0.04 ± 0.01	-0.05 ± 0.02	-0.03 ± 0.03
103	Tilt (RNA) (103)	-0.3 ± 0.14	-0.3 ± 0.14	0.16 ± 0.12	-0.1 ± 0.05	0.06 ± 0.01	0.06 ± 0.01	0.11 ± 0.02	0.11 ± 0.02	0.02 ± 0.01	0.14 ± 0.03	0.14 ± 0.03	0.02 ± 0.01	-0.01 ± 0.01	-0.01 ± 0.01	-0.05 ± 0.02	-0.04 ± 0.04
70	Tilt stiffness (70)	0.01 ± 0.0	0.01 ± 0.0	0.0 ± 0.0	-0.03 ± 0.01	0.01 ± 0.0	0.01 ± 0.0	-0.02 ± 0.0	-0.02 ± 0.0	0.0 ± 0.0	0.01 ± 0.0	0.01 ± 0.0	0.0 ± 0.0	0.01 ± 0.0	0.01 ± 0.0	0.0 ± 0.0	-0.01 ± 0.01
56	Tilt_rise (56)	0.29 ± 0.04	0.29 ± 0.04	-0.08 ± 0.03	-0.05 ± 0.02	-0.1 ± 0.02	-0.1 ± 0.02	-0.04 ± 0.0	-0.04 ± 0.0	-0.12 ± 0.02	0.05 ± 0.02	0.05 ± 0.02	-0.12 ± 0.02	0.04 ± 0.02	0.04 ± 0.02	0.02 ± 0.02	-0.04 ± 0.03
44	Tilt_roll (44)	0.58 ± 8.37	0.56 ± 7.43	0.03 ± 1.19	-0.08 ± 0.24	0.45 ± 10.57	0.41 ± 8.57	0.13 ± 4.28	0.12 ± 3.49	-0.64 ± 12.67	-0.11 ± 1.31	-0.11 ± 1.19	-0.65 ± 12.95	-0.38 ± 11.04	-0.36 ± 10.16	-0.32 ± 7.74	0.15 ± 4.74
54	Tilt_shift (54)	-0.1 ± 0.04	-0.1 ± 0.04	-0.19 ± 0.89	-0.15 ± 0.75	-0.03 ± 0.05	-0.03 ± 0.05	-0.09 ± 0.05	-0.09 ± 0.05	0.03 ± 0.19	0.06 ± 0.3	0.06 ± 0.3	0.03 ± 0.19	0.2 ± 0.3	0.2 ± 0.29	0.12 ± 0.18	0.09 ± 0.13
55	Tilt_slide (55)	0.15 ± 0.02	0.15 ± 0.02	0.19 ± 0.03	-0.17 ± 0.06	0.2 ± 0.05	0.2 ± 0.05	-0.01 ± 0.01	-0.01 ± 0.01	-0.23 ± 0.06	-0.01 ± 0.01	-0.01 ± 0.01	-0.23 ± 0.06	-0.14 ± 0.07	-0.14 ± 0.07	0.3 ± 0.21	-0.17 ± 0.17
40	Tilt_tilt (40)	0.01 ± 0.0	0.01 ± 0.0	0.01 ± 0.0	-0.02 ± 0.01	0.01 ± 0.0	0.01 ± 0.0	-0.02 ± 0.0	-0.02 ± 0.0	0.0 ± 0.0	0.0 ± 0.0	0.0 ± 0.0	0.0 ± 0.0	0.01 ± 0.0	0.01 ± 0.0	0.0 ± 0.0	-0.01 ± 0.01
5	Tip (5)	0.01 ± 0.01	0.01 ± 0.01	0.02 ± 0.01	0.23 ± 0.05	0.02 ± 0.01	0.02 ± 0.01	-0.16 ± 0.03	-0.16 ± 0.03	-0.03 ± 0.01	-0.01 ± 0.01	-0.01 ± 0.01	-0.03 ± 0.01	-0.03 ± 0.01	-0.03 ± 0.01	0.04 ± 0.04	-0.03 ± 0.03
1	Twist (1)	0.01 ± 0.0	0.01 ± 0.0	-0.01 ± 0.0	-0.01 ± 0.0	-0.01 ± 0.0	-0.01 ± 0.0	0.01 ± 0.0	0.01 ± 0.0	-0.01 ± 0.0	0.01 ± 0.0	0.01 ± 0.0	-0.01 ± 0.0	-0.0 ± 0.0	-0.0 ± 0.0	0.0 ± 0.0	-0.0 ± 0.0
118	Twist (118)	0.0 ± 0.0	0.0 ± 0.0	0.0 ± 0.0	-0.0 ± 0.0	-0.0 ± 0.0	-0.0 ± 0.0	-0.0 ± 0.0	-0.0 ± 0.0	-0.0 ± 0.0	0.0 ± 0.0	0.0 ± 0.0	-0.0 ± 0.0	-0.0 ± 0.0	-0.0 ± 0.0	-0.0 ± 0.0	-0.0 ± 0.0
120	Twist (120)	0.0 ± 0.0	0.0 ± 0.0	0.01 ± 0.0	-0.0 ± 0.0	-0.0 ± 0.0	-0.0 ± 0.0	-0.01 ± 0.0	-0.01 ± 0.0	-0.0 ± 0.0	0.0 ± 0.0	0.0 ± 0.0	-0.0 ± 0.0	-0.0 ± 0.0	-0.0 ± 0.0	0.0 ± 0.0	-0.0 ± 0.0
61	Twist (61)	-0.0 ± 0.0	-0.0 ± 0.0	-0.01 ± 0.0	0.01 ± 0.0	-0.01 ± 0.0	-0.01 ± 0.0	0.01 ± 0.0	0.01 ± 0.0	-0.01 ± 0.0	0.01 ± 0.0	0.01 ± 0.0	-0.01 ± 0.0	-0.0 ± 0.0	-0.0 ± 0.0	0.01 ± 0.0	-0.0 ± 0.0
88	Twist (88)	0.0 ± 0.0	0.0 ± 0.0	-0.0 ± 0.0	0.01 ± 0.0	0.0 ± 0.0	0.0 ± 0.0	0.0 ± 0.0	0.0 ± 0.0	-0.01 ± 0.0	0.01 ± 0.0	0.01 ± 0.0	-0.01 ± 0.0	-0.0 ± 0.0	-0.0 ± 0.0	0.0 ± 0.0	-0.0 ± 0.0
92	Twist (92)	-0.0 ± 0.0	-0.0 ± 0.0	-0.01 ± 0.0	0.01 ± 0.0	-0.0 ± 0.0	-0.0 ± 0.0	0.01 ± 0.0	0.01 ± 0.0	-0.01 ± 0.0	0.01 ± 0.0	0.01 ± 0.0	-0.01 ± 0.0	-0.0 ± 0.0	-0.0 ± 0.0	0.0 ± 0.0	0.0 ± 0.0
98	Twist (98)	0.0 ± 0.0	0.0 ± 0.0	-0.01 ± 0.0	0.0 ± 0.0	0.0 ± 0.0	0.0 ± 0.0	0.0 ± 0.0	0.0 ± 0.0	-0.01 ± 0.0	0.01 ± 0.0	0.01 ± 0.0	-0.01 ± 0.0	-0.0 ± 0.0	-0.0 ± 0.0	0.01 ± 0.0	-0.0 ± 0.0
26	Twist (DNA-protein complex) (26)	0.0 ± 0.0	0.0 ± 0.0	-0.01 ± 0.0	0.01 ± 0.0	-0.0 ± 0.0	-0.0 ± 0.0	0.0 ± 0.0	0.0 ± 0.0	-0.0 ± 0.0	0.0 ± 0.0	0.0 ± 0.0	-0.0 ± 0.0	-0.0 ± 0.0	-0.0 ± 0.0	0.0 ± 0.0	0.0 ± 0.0
37	Twist (DNA-protein complex) (37)	0.0 ± 0.0	0.0 ± 0.0	-0.01 ± 0.0	0.01 ± 0.0	-0.0 ± 0.0	-0.0 ± 0.0	0.01 ± 0.0	0.01 ± 0.0	-0.0 ± 0.0	0.0 ± 0.0	0.0 ± 0.0	-0.0 ± 0.0	-0.0 ± 0.0	-0.0 ± 0.0	-0.0 ± 0.0	0.0 ± 0.0
105	Twist (RNA) (105)	-0.0 ± 0.0	-0.0 ± 0.0	0.0 ± 0.0	0.0 ± 0.0	0.0 ± 0.0	0.0 ± 0.0	-0.0 ± 0.0	-0.0 ± 0.0	-0.0 ± 0.0	0.0 ± 0.0	0.0 ± 0.0	-0.0 ± 0.0	0.0 ± 0.0	0.0 ± 0.0	0.01 ± 0.0	-0.0 ± 0.0
71	Twist stiffness (71)	0.0 ± 0.0	0.0 ± 0.0	0.02 ± 0.01	-0.02 ± 0.01	0.02 ± 0.0	0.02 ± 0.0	-0.03 ± 0.0	-0.03 ± 0.0	0.01 ± 0.0	-0.0 ± 0.0	-0.0 ± 0.0	0.01 ± 0.0	0.0 ± 0.0	0.0 ± 0.0	-0.0 ± 0.0	-0.02 ± 0.01
53	Twist_rise (53)	0.02 ± 0.01	0.02 ± 0.01	-0.03 ± 0.01	-0.02 ± 0.01	0.01 ± 0.0	0.01 ± 0.0	-0.0 ± 0.0	-0.0 ± 0.0	0.01 ± 0.0	-0.0 ± 0.0	-0.0 ± 0.0	0.01 ± 0.0	-0.01 ± 0.0	-0.01 ± 0.0	0.01 ± 0.0	-0.0 ± 0.0
43	Twist_roll (43)	0.02 ± 0.0	0.02 ± 0.0	0.01 ± 0.0	0.01 ± 0.0	0.01 ± 0.0	0.01 ± 0.0	-0.02 ± 0.0	-0.02 ± 0.0	-0.01 ± 0.0	0.02 ± 0.0	0.02 ± 0.0	-0.01 ± 0.0	-0.02 ± 0.0	-0.02 ± 0.0	0.0 ± 0.0	-0.02 ± 0.02
51	Twist_shift (51)	0.59 ± 0.09	0.59 ± 0.09	-0.15 ± 0.04	-0.1 ± 0.04	-0.01 ± 0.0	-0.01 ± 0.0	-0.16 ± 0.04	-0.16 ± 0.04	-0.32 ± 0.1	-0.07 ± 0.01	-0.07 ± 0.01	-0.33 ± 0.1	0.12 ± 0.07	0.12 ± 0.07	-0.06 ± 0.03	0.08 ± 0.09
52	Twist_slide (52)	0.03 ± 0.01	0.03 ± 0.01	-0.02 ± 0.01	-0.03 ± 0.01	0.01 ± 0.0	0.01 ± 0.0	-0.06 ± 0.01	-0.06 ± 0.01	0.01 ± 0.0	0.02 ± 0.0	0.02 ± 0.0	0.01 ± 0.0	0.02 ± 0.01	0.02 ± 0.01	0.01 ± 0.0	-0.02 ± 0.01
42	Twist_tilt (42)	0.65 ± 0.12	0.65 ± 0.12	-0.1 ± 0.03	-0.12 ± 0.04	0.01 ± 0.03	0.01 ± 0.03	-0.11 ± 0.04	-0.11 ± 0.04	-0.31 ± 0.15	-0.01 ± 0.02	-0.01 ± 0.02	-0.31 ± 0.16	-0.08 ± 0.05	-0.08 ± 0.05	-0.03 ± 0.01	0.06 ± 0.11
39	Twist_twist (39)	0.01 ± 0.0	0.01 ± 0.0	0.01 ± 0.0	-0.01 ± 0.0	0.01 ± 0.0	0.01 ± 0.0	-0.03 ± 0.0	-0.03 ± 0.0	0.01 ± 0.0	0.0 ± 0.0	0.0 ± 0.0	0.01 ± 0.0	0.01 ± 0.0	0.01 ± 0.0	0.0 ± 0.0	-0.02 ± 0.01
99	Wedge (99)	0.06 ± 0.02	0.06 ± 0.02	-0.03 ± 0.01	-0.05 ± 0.02	-0.04 ± 0.0	-0.04 ± 0.0	-0.01 ± 0.0	-0.01 ± 0.0	0.05 ± 0.01	0.01 ± 0.0	0.01 ± 0.0	0.05 ± 0.01	-0.03 ± 0.01	-0.03 ± 0.01	0.01 ± 0.0	0.02 ± 0.02

Table S5: Significance levels of DNA property changes induced by dinucleotide contents

property id	property name	AA	TT	AT	TA	AC	GT	CA	TG	AG	TC	GA	CT	CC	GG	GC	CG
79	Adenine content (79)	8.95	-3.54	11.95	6.78	2.19	-9.48	2.42	-8.79	2.42	-8.85	2.16	-7.08	-3.15	-3.17	-2.16	-1.15
4	Bend (4)	-0.65	-0.65	-2.01	3.06	-1.38	-1.38	3.02	3.01	-6.69	-7.55	-7.4	-6.78	-4.1	-4.12	-0.08	-2.01
21	Clash Strength (21)	-4.97	-5.02	2.03	-2.54	-1.58	-1.58	-4.03	-4.02	7.53	-8.53	-8.48	7.64	5.33	5.35	-2.03	1.34
81	Cytosine content (81)	-3.52	-3.54	-3.09	-2.54	3.45	-9.48	3.35	-8.79	-7.07	3.57	-8.77	3.79	7.12	-3.17	8.67	1.91

100	Direction (100)	-0.53	0.53	-2.8	-2.37	1.02	-1.16	-1.32	1.06	-2.68	-1.32	1.09	-4.89	-2.53	1.89	3.81	-1.23
123	Enthalpy (123)	-7.39	-7.5	-5.21	-3.64	2.05	2.05	2.79	2.8	-2.19	0.55	0.55	-2.17	-0.77	-0.78	3.01	1.3
22	Enthalpy (22)	-1.29	-1.29	-4.03	-3.68	3.2	3.2	-0.59	-0.59	-4.48	1.33	1.33	-4.44	5.17	5.18	4.3	1.44
110	Enthalpy (RNA) (110)	-5.5	-5.57	-4.12	-3.11	2.72	2.72	3.34	-3.52	-3.35	2.81	8.5	-3.32	5.64	5.66	3.18	-0.8
114	Enthalpy (RNA) (114)	-4.75	-4.8	-2.93	-3.62	2.21	2.21	0.15	0.15	0.23	4.57	4.53	0.22	5.93	5.94	3.47	-0.04
124	Entropy (124)	-7.1	-7.2	-4.32	-4.61	3.61	3.61	5.18	5.2	-4.16	2.17	2.17	-4.12	-2.61	-2.62	2.67	1.22
23	Entropy (23)	0.05	0.05	-3.9	-3.83	4.9	4.89	-1.61	-1.61	-5.29	2.11	2.11	-5.25	4.86	4.87	4.39	1.44
111	Entropy (RNA) (111)	-5.58	-5.65	-3.96	-0.76	3.26	3.26	4.75	-4.67	-4.26	3.4	10.21	-4.23	5.51	5.53	2.98	-0.94
115	Entropy (RNA) (115)	-4.7	-4.75	-0.01	-3.44	2.62	2.62	-0.17	-0.17	0.03	6.01	5.93	0.03	5.98	5.98	3.27	-0.45
122	Flexibility_shift (122)	-3.19	-3.21	-3.16	-3.37	11.08	11.02	-4.97	-4.97	7.15	-2.17	-2.18	7.25	-1.42	-1.43	-2.12	1.3
121	Flexibility_slide (121)	6.58	6.68	7.87	-1.45	1.81	1.81	-8.41	-8.43	-1.75	2.42	2.44	-1.74	-2.48	-2.49	-2.6	-1.26
125	Free energy (125)	-6.78	-6.87	-5.43	-2.94	1.22	1.22	1.38	1.38	-0.51	-0.26	-0.26	-0.51	5.94	5.92	3.94	1.56
34	Free energy (34)	-5.34	-5.4	-4.5	-3.67	1.88	1.88	0.96	0.96	0.74	0.09	0.09	0.74	5.97	5.95	4.3	1.58
35	Free energy (35)	0.27	0.27	-6.26	-2.94	-4.48	-4.47	1.16	1.15	-2.85	-2.15	-2.17	-2.82	5.31	5.31	3.98	1.47
36	Free energy (36)	-6.31	-6.4	-4.96	-3.54	1.48	1.48	1.78	1.78	-1.02	-0.94	0.22	-1.02	5.85	5.84	3.91	1.52
38	Free energy (38)	-5.36	-5.42	-3.79	-2.85	1.57	1.57	1.52	1.52	0.06	1.1	1.1	0.06	5.31	5.29	4.92	1.65
72	Free energy (72)	-6.81	-6.9	-4.43	-3.29	-0.3	-0.3	1.67	1.67	-0.32	-0.3	-0.3	-0.32	5.82	5.81	4.52	1.48
73	Free energy (73)	-6.78	-6.87	-5.43	-2.94	1.22	1.22	1.38	1.38	-0.51	-0.26	-0.26	-0.51	5.94	5.92	3.94	1.56
74	Free energy (74)	-6.42	-6.51	-4.79	-3.46	0.59	0.59	3.46	3.47	-2.74	0.94	0.95	-2.71	5.73	5.71	3.62	1.56
75	Free energy (75)	-6.4	-6.5	-5.1	-3.13	-0.24	-0.24	2.97	2.97	0.13	0.34	0.35	0.12	6.03	6.03	4.63	1.62
112	Free energy (RNA) (112)	-4.87	-4.92	-4.4	-3.8	2.02	2.02	0.24	-0.46	-0.5	2.05	3.16	-0.5	6.35	6.35	3.92	0.78
113	Free energy (RNA) (113)	-4.59	-4.63	-4.71	-4.04	1.47	1.47	0.79	0.79	0.62	2.09	2.09	0.61	6.33	6.33	4.3	1.65
76	GC content (76)	-3.52	-3.54	-3.09	-2.54	0.88	0.88	0.91	0.91	0.97	0.88	0.88	0.97	6.8	6.77	8.68	1.91
80	Guanine content (80)	-3.52	-3.54	-3.09	-2.54	-9.54	3.46	-8.78	3.37	3.79	-8.85	3.57	-7.08	-3.15	7.11	8.52	1.91
29	Hydrophilicity (RNA) (29)	-3.57	3.23	-3.27	-2.74	-8.27	-2.79	-7.62	8.77	-7.22	10.55	-8.46	7.86	3.52	-3.07	-1.66	1.44
31	Hydrophilicity (RNA) (31)	-3.6	2.77	-3.57	-3.92	-7.38	1.36	-4.55	6.59	-6.88	11.39	-7.79	7.79	4.02	-2.62	-0.08	1.43
6	Inclination (6)	-0.0	0.0	-0.11	-0.11	-0.01	0.01	0.01	-0.01	-0.01	0.01	-0.01	0.01	-0.01	0.01	-0.07	-0.06
78	Keto (GT) content (78)	-3.56	3.74	3.9	3.06	-9.59	9.0	-8.04	6.11	-6.6	6.54	6.43	5.23	-3.12	3.08	2.1	1.15
8	Major Groove Depth (8)	6.11	6.16	-0.35	3.12	5.18	5.17	-5.83	-5.85	-0.04	-4.86	-4.82	-0.04	-4.2	-4.22	-3.9	-1.96
10	Major Groove Distance (10)	-2.48	-2.49	-3.09	2.55	-9.55	-9.49	8.74	8.75	-5.89	1.45	1.44	-5.9	-2.77	-2.78	-2.16	1.14
9	Major Groove Size (9)	0.1	0.1	3.09	-2.54	0.1	0.1	0.1	0.1	7.08	-8.83	-8.75	7.09	0.1	0.1	-2.15	1.15
7	Major Groove Width (7)	-6.48	-6.58	-3.83	-4.26	-3.62	-3.62	1.19	1.19	0.86	2.99	2.99	0.85	4.6	4.61	4.43	1.69
108	Melting Temperature (108)	-7.46	-7.58	-7.7	-3.29	7.52	7.5	-3.68	-3.67	-2.86	4.85	4.81	-2.84	5.65	5.63	2.64	-0.06
16	Melting Temperature (16)	-6.54	-6.63	-6.95	-2.95	6.24	6.23	-4.22	-4.22	-3.18	4.28	4.25	-3.15	5.57	5.55	3.04	-0.03
12	Minor Groove Depth (12)	2.3	2.31	-2.97	0.6	-8.55	-8.51	6.86	6.87	-1.55	7.58	7.51	-1.56	-0.55	-0.55	-1.35	1.18
14	Minor Groove Distance (14)	-3.51	-3.54	3.1	-2.55	9.97	9.91	-8.47	-8.49	-6.83	-8.88	-8.79	-6.85	-3.14	-3.15	2.19	-1.15
13	Minor Groove Size (13)	-6.57	-6.67	-2.26	-3.24	-1.69	-1.69	3.5	3.51	5.81	-3.91	-3.93	5.89	5.42	5.43	-0.99	1.32
11	Minor Groove Width (11)	0.84	0.84	0.96	3.02	5.79	5.78	-6.61	-6.64	-0.73	-9.53	-9.27	-0.72	-4.26	-4.28	-3.26	-0.36
18	Mobility to bend towards major groove (18)	4.85	5.57	6.26	0.67	0.03	-4.66	0.01	-3.32	0.03	-3.54	1.72	-2.28	-4.83	-5.17	-3.14	-1.78
19	Mobility to bend towards minor groove (19)	-6.74	-6.84	-5.37	-4.36	-1.52	-0.99	2.08	5.75	-2.14	4.94	-0.44	2.17	4.85	5.15	4.19	1.48
15	Persistance Length (15)	-6.43	-6.52	-3.9	-3.01	0.32	0.32	0.36	0.36	0.34	0.31	0.31	0.34	6.24	6.26	7.68	1.98

17	Probability contacting nucleosome core (17)	4.94	5.0	-2.62	-2.3	-3.53	-3.52	4.95	4.93	3.89	-0.54	-0.54	3.83	-4.09	-4.08	-2.29	-1.11
20	Propeller Twist (20)	5.17	5.23	4.78	-1.77	-10.04	-9.97	-9.03	-9.07	3.14	3.81	3.83	3.12	0.16	0.16	-6.56	-1.54
77	Purine (AG) content (77)	3.53	-3.54	0.05	0.05	0.07	0.07	0.07	0.07	7.11	-8.85	8.86	-7.08	-3.15	3.17	0.06	0.06
3	Rise (3)	-5.18	-5.24	2.35	-3.53	-1.84	-1.83	-4.94	-4.93	3.32	-0.61	-0.62	3.33	4.15	4.17	3.62	1.71
66	Rise (66)	-5.49	-5.55	-5.51	1.87	-6.19	-6.17	-0.99	-0.99	-4.61	9.9	9.64	-4.57	4.44	4.45	2.39	-0.99
97	Rise (97)	-5.68	-5.75	-5.05	2.01	-3.86	-3.85	5.86	5.87	-3.93	-3.67	-3.69	-3.89	4.76	4.77	2.54	1.49
32	Rise (DNA-protein complex) (32)	-4.76	-4.81	-4.28	1.48	3.54	3.54	3.42	3.43	3.61	3.65	3.63	3.63	4.88	4.87	5.62	1.85
87	Rise (DNA-protein complex) (87)	-4.68	-4.73	-5.39	2.06	1.22	1.22	-2.27	-2.27	-1.23	2.62	2.61	-1.23	4.45	4.47	3.67	1.62
102	Rise (RNA) (102)	-4.71	-4.75	-0.74	1.22	-0.93	-0.93	-7.59	-7.59	6.2	10.54	10.29	6.28	4.19	4.21	-1.35	1.42
107	Rise stiffness (107)	-6.72	-6.8	2.91	-2.62	10.47	10.39	-8.39	-8.39	-6.45	10.34	10.14	-6.43	4.21	4.23	2.26	-1.12
47	Rise_rise (47)	-0.87	-0.87	3.2	-2.51	8.85	8.8	-8.98	-9.0	-7.19	6.98	6.98	-7.22	-4.12	-4.13	2.07	-1.15
116	Roll (116)	0.02	0.02	0.02	-0.02	0.01	0.01	-0.01	-0.01	-0.01	-0.01	-0.01	-0.01	-0.01	-0.01	0.01	-0.02
119	Roll (119)	1.09	1.1	-2.62	2.08	-11.5	-11.42	9.04	9.1	-6.59	12.38	11.94	-6.6	-2.51	-2.52	-2.26	1.29
63	Roll (63)	0.97	0.97	-2.32	-2.12	1.6	1.6	-7.75	-7.77	7.43	-5.87	-5.92	7.52	5.52	5.59	-2.49	1.37
90	Roll (90)	-4.99	-5.04	-2.89	1.58	-5.39	-5.37	-1.56	-1.55	5.03	-7.17	-7.19	5.08	5.58	5.63	-2.51	1.38
94	Roll (94)	-4.15	-4.19	-2.73	1.74	-3.72	-3.72	-4.83	-4.8	10.0	-5.8	-5.8	10.13	4.84	4.89	-2.03	1.2
25	Roll (DNA-protein complex) (25)	-3.86	-3.89	-3.03	0.57	-9.39	-9.33	8.9	8.96	8.58	-0.99	-0.99	8.74	7.72	7.69	-2.28	1.3
84	Roll (DNA-protein complex) (84)	-3.77	-3.8	-3.72	1.47	-8.18	-8.13	7.49	7.53	7.23	-3.47	-3.48	7.35	6.46	6.47	-2.1	1.34
104	Roll (RNA) (104)	-4.62	-4.66	-4.18	2.21	-8.38	-8.33	7.3	7.34	0.83	7.63	7.5	0.83	2.45	3.77	-1.92	1.25
69	Roll stiffness (69)	2.38	2.38	2.91	-2.59	10.24	10.17	-8.46	-8.47	-5.9	6.86	6.74	-5.87	-2.67	-2.69	2.22	-1.13
59	Roll_rise (59)	0.05	0.05	-0.05	0.05	-0.04	-0.04	0.04	0.04	0.02	0.04	0.04	0.02	-0.06	-0.06	-0.04	0.03
41	Roll_roll (41)	3.03	3.04	2.95	-2.56	10.08	10.01	-8.6	-8.6	5.99	-6.34	-6.35	6.07	3.57	3.58	2.23	-1.12
57	Roll_shift (57)	4.85	4.89	5.29	-3.27	2.81	2.82	-3.38	-3.4	-2.76	2.65	2.67	-2.75	-1.65	-1.65	-1.85	0.89
58	Roll_slide (58)	8.24	8.3	6.98	-1.73	-9.2	-9.15	-6.08	-6.07	3.19	-9.84	-9.8	3.15	-5.22	-5.22	-1.89	0.78
64	Shift (64)	0.12	0.12	-0.12	0.12	-0.11	-0.11	-0.11	-0.11	-0.11	-0.14	-0.14	-0.11	-0.11	-0.11	0.11	-0.11
95	Shift (95)	-0.15	-0.15	-0.15	-0.13	-0.16	-0.16	-0.18	-0.18	0.21	-0.24	-0.24	0.21	-0.24	-0.24	-0.25	-0.22
30	Shift (DNA-protein complex) (30)	1.14	1.13	-1.88	-1.76	-0.51	-0.51	-1.03	-1.02	-0.57	0.66	0.66	-0.57	-0.62	-0.62	-0.46	-0.39
85	Shift (DNA-protein complex) (85)	0.03	0.03	0.02	0.02	-0.03	-0.03	-0.03	-0.03	-0.03	0.03	0.03	-0.03	-0.03	-0.03	0.02	0.03
24	Shift (RNA) (24)	-0.66	-0.66	-0.66	-1.02	0.9	0.9	0.68	0.68	-1.95	0.48	0.48	-1.96	-2.43	-2.43	0.9	1.49
68	Shift stiffness (68)	3.52	3.54	-3.09	-2.52	9.15	9.1	-8.85	-8.85	7.34	8.83	8.75	7.34	3.18	3.2	-2.1	-1.13
49	Shift_rise (49)	3.81	3.84	-3.14	-2.54	-5.08	-5.06	-6.63	-6.61	-6.18	-3.55	-3.58	-6.11	3.47	3.49	2.22	-1.03
45	Shift_shift (45)	3.47	3.49	-3.1	-2.52	-8.35	-8.3	-1.5	-1.49	4.79	-7.32	-7.32	4.84	3.36	3.38	2.47	-1.08
48	Shift_slide (48)	-0.08	-0.08	0.07	-0.08	0.09	0.09	0.1	0.1	0.07	-0.12	-0.12	0.07	-0.15	-0.15	-0.16	0.13
65	Slide (65)	-2.27	-2.28	-1.47	0.62	-6.94	-6.93	4.15	4.17	1.5	-5.98	-5.93	1.5	0.67	0.67	1.28	1.55
91	Slide (91)	-3.3	-3.33	-2.41	1.47	-9.26	-9.2	8.11	8.15	3.1	-6.91	-6.84	3.12	8.47	8.41	-0.83	1.53
96	Slide (96)	-3.11	-3.12	-2.37	1.45	-9.6	-9.53	6.97	7.01	0.72	-6.36	-6.32	0.72	8.39	8.34	0.27	1.63
28	Slide (DNA-protein complex) (28)	-0.21	-0.2	0.16	-0.33	0.09	0.09	-0.15	-0.15	0.13	-0.09	-0.09	0.12	0.06	0.06	0.04	-0.02
86	Slide (DNA-protein complex) (86)	-1.94	-1.94	4.77	-2.89	4.43	4.42	-5.97	-5.95	4.32	-4.99	-5.0	4.26	2.14	2.15	1.52	-0.94
101	Slide (RNA) (101)	-5.34	-5.4	-7.23	-1.53	-1.98	-1.98	-1.21	-1.21	0.08	7.14	7.02	0.08	4.8	4.82	-1.31	1.35
67	Slide stiffness (67)	1.42	1.42	3.48	-2.45	7.23	7.2	-8.39	-8.43	-5.42	-9.88	-9.6	-5.48	-3.6	-3.61	1.72	-1.5
50	Slide_rise (50)	-3.68	-3.71	3.08	-2.57	10.43	10.36	-8.28	-8.3	-6.58	-8.77	-8.69	-6.58	-3.03	-3.04	2.27	-1.02
46	Slide_slide (46)	4.27	4.3	3.35	-2.42	7.44	7.41	-9.48	-9.49	-3.63	-10.34	-10.19	-3.66	3.01	3.02	-5.21	-1.14

109	Stacking energy (109)	-5.34	-5.4	-4.23	-3.14	7.1	7.09	-3.96	-3.95	-3.83	6.1	6.02	-3.8	0.25	0.25	2.94	1.99
2	Stacking energy (2)	3.45	3.47	-3.1	-2.57	8.34	8.29	8.82	8.83	-3.7	-7.36	-7.27	-3.72	-3.16	-3.18	2.15	1.14
33	Stacking energy (33)	-5.43	-5.5	-4.68	-2.93	5.76	5.75	-2.85	-2.84	-2.32	4.85	4.81	-2.3	1.25	1.26	3.14	1.85
60	Stacking energy (60)	6.04	6.13	-1.29	1.81	6.16	6.14	7.71	7.7	-6.76	-10.33	-10.11	-6.84	-3.6	-3.62	-2.44	1.07
106	Stacking energy (RNA) (106)	-2.35	-2.36	4.06	2.91	-5.46	-5.45	2.0	2.0	-2.77	-0.13	-0.13	-2.78	-3.36	-3.37	1.99	1.05
82	Thymine content (82)	-3.52	9.02	11.73	6.72	-9.54	2.21	-8.78	2.45	-7.07	2.16	-8.77	2.42	-3.15	-3.17	-2.16	-1.15
117	Tilt (117)	-0.02	0.02	-0.16	-0.15	-0.02	0.02	0.02	-0.02	-0.02	0.02	-0.02	0.02	0.02	-0.02	-0.14	-0.15
62	Tilt (62)	-0.0	-0.0	0.0	0.0	-0.01	-0.01	-0.0	-0.0	0.01	0.0	0.0	0.01	0.01	0.01	0.01	0.01
89	Tilt (89)	-5.0	-5.05	-2.83	-2.36	5.73	5.72	-11.27	-11.26	14.78	-3.52	-3.53	15.03	5.33	5.38	-1.95	-1.05
93	Tilt (93)	-3.7	-3.71	-2.94	-2.41	-5.34	-5.32	-9.98	-9.96	9.43	1.87	1.87	9.62	6.41	6.48	-1.8	-1.0
27	Tilt (DNA-protein complex) (27)	4.6	4.65	-2.96	-2.42	-8.18	-7.31	-9.19	-9.2	4.95	5.15	5.19	4.86	1.71	1.72	-2.0	-1.1
83	Tilt (DNA-protein complex) (83)	5.72	5.79	-2.85	-2.36	-7.98	-7.94	-7.11	-7.1	4.85	4.36	4.4	4.76	-3.37	-3.39	-2.1	-1.15
103	Tilt (RNA) (103)	-2.19	-2.2	1.31	-2.04	4.03	4.04	4.69	4.71	1.96	5.72	5.67	1.96	-1.66	-1.66	-2.22	-1.17
70	Tilt stiffness (70)	2.71	2.72	1.95	-2.6	9.64	9.59	-8.12	-8.11	6.73	10.6	10.29	6.82	3.73	3.76	4.49	-1.08
56	Tilt_rise (56)	7.1	7.23	-3.12	-2.38	-5.39	-5.37	-8.08	-8.07	-5.37	2.82	2.84	-5.3	1.8	1.81	1.11	-1.07
44	Tilt_roll (44)	0.07	0.08	0.03	-0.33	0.04	0.05	0.03	0.03	-0.05	-0.09	-0.09	-0.05	-0.03	-0.04	-0.04	0.03
54	Tilt_shift (54)	-2.19	-2.21	-0.21	-0.2	-0.59	-0.59	-1.81	-1.81	0.18	0.21	0.21	0.18	0.68	0.68	0.66	0.68
55	Tilt_slide (55)	7.92	8.09	6.41	-3.09	3.68	3.69	-1.19	-1.2	-4.22	-0.57	-0.57	-4.22	-1.99	-2.0	1.47	-0.99
40	Tilt_tilt (40)	2.98	2.99	2.77	-2.59	10.52	10.44	-8.51	-8.51	5.07	10.81	10.54	5.12	3.51	3.53	2.48	-1.11
5	Tip (5)	1.32	1.32	2.06	4.26	1.76	1.76	-5.67	-5.67	-4.67	-0.75	-0.75	-4.72	-4.18	-4.2	1.21	-1.21
1	Twist (1)	3.74	3.77	-2.92	-2.45	-9.82	-9.76	8.63	8.63	-7.18	8.23	8.19	-7.21	-3.67	-3.68	1.98	-1.18
118	Twist (118)	5.41	5.47	7.09	-0.01	-4.42	-4.42	-1.88	-1.88	-0.12	0.89	0.89	-0.12	-4.83	-4.85	-3.77	-1.62
120	Twist (120)	5.31	5.37	3.72	-2.01	-2.23	-2.23	-9.17	-9.21	-3.09	5.8	5.8	-3.1	-5.89	-5.88	1.63	-1.27
61	Twist (61)	-2.98	-2.99	-3.02	2.59	-10.03	-9.96	8.7	8.7	-7.14	8.22	8.17	-7.16	-3.82	-3.83	2.07	-1.16
88	Twist (88)	1.98	1.98	-2.54	2.81	1.41	1.41	5.2	5.19	-7.29	6.67	6.67	-7.33	-4.03	-4.05	1.82	-1.21
92	Twist (92)	-2.71	-2.72	-3.06	2.57	-9.89	-9.83	8.59	8.6	-7.02	8.64	8.58	-7.03	-3.26	-3.28	2.05	1.09
98	Twist (98)	3.98	4.01	-2.86	2.79	4.61	4.6	5.2	5.19	-7.19	7.84	7.79	-7.2	-4.65	-4.67	2.12	-1.16
26	Twist (DNA-protein complex) (26)	4.47	4.51	-2.92	2.69	-10.72	-10.64	6.92	7.01	-7.56	6.37	6.37	-7.61	-4.29	-4.31	1.42	0.98
37	Twist (DNA-protein complex) (37)	3.88	3.91	-3.04	2.6	-10.0	-9.93	8.47	8.48	-7.12	8.05	8.0	-7.15	-3.39	-3.4	-2.54	1.11
105	Twist (RNA) (105)	-2.84	-2.86	3.31	2.92	6.68	6.66	-8.15	-8.17	-7.36	6.21	6.19	-7.38	2.78	2.79	2.11	-1.15
71	Twist stiffness (71)	3.85	3.81	3.31	-2.48	8.26	8.21	-9.06	-9.08	6.68	-3.82	-3.8	6.63	1.03	1.04	-4.24	-1.15
53	Twist_rise (53)	3.2	3.22	-3.17	-2.6	10.84	10.76	-6.08	-6.08	8.39	-4.99	-4.99	8.45	-2.97	-2.98	2.25	-1.09
43	Twist_roll (43)	8.83	9.01	9.9	4.74	2.01	2.01	-7.13	-7.17	-4.52	3.39	3.41	-4.56	-4.06	-4.08	0.54	-1.23
51	Twist_shift (51)	6.64	6.73	-4.06	-2.98	-2.73	-2.73	-3.66	-3.66	-3.15	-4.79	-4.83	-3.12	1.76	1.78	-1.68	0.9
52	Twist_slide (52)	3.3	3.32	-3.24	-2.58	9.81	9.75	-8.56	-8.56	6.54	10.29	10.1	6.61	3.48	3.5	2.49	-1.11
42	Twist_tilt (42)	5.29	5.22	-3.71	-3.46	0.29	0.29	-2.89	-2.89	-2.04	-0.37	-0.38	-2.01	-1.6	-1.62	-3.4	0.56
39	Twist_twist (39)	4.53	4.57	4.0	-2.21	7.07	7.04	-9.04	-9.05	5.01	3.13	3.14	4.97	2.75	2.77	1.38	-1.15
99	Wedge (99)	3.67	3.7	-2.9	-2.45	-9.19	-9.14	-8.49	-8.52	7.05	7.35	7.35	7.04	-3.21	-3.23	1.81	1.12

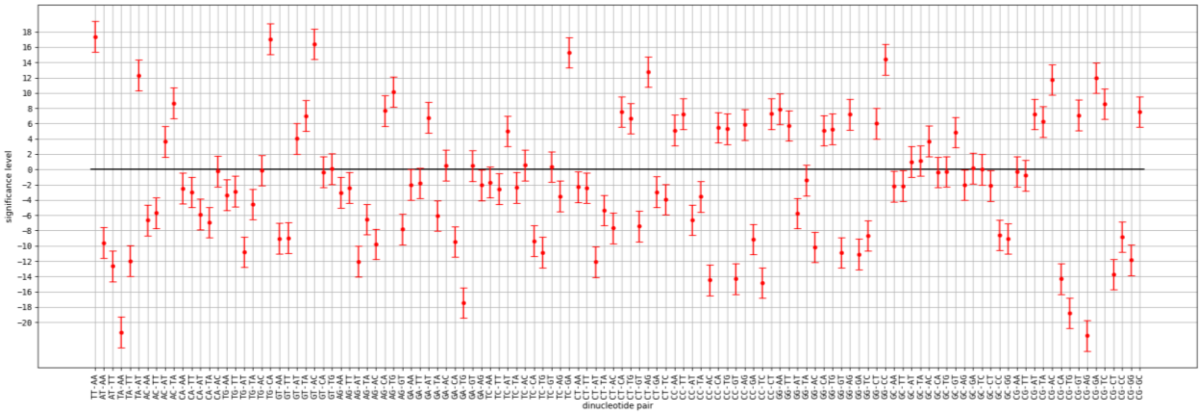

Figure S1: Significance levels of dimer-pairs: Significance levels for correlations (see 2.1) of all dinucleotide pairs (x-axis) in relation to Chargaff model. Deviations from zero larger than 1.0 were not expected by the model and are thus considered significant.
